# CRISPRa Lipid Nanocomplex‐Mediated Mt3 Targeting Enhances Astrocytic Endocytosis of Amyloid‐β in an Alzheimer's Disease Mouse Model

**DOI:** 10.1002/advs.202503725

**Published:** 2025-12-12

**Authors:** Junhang Park, Boyoung Kim, Minki Ha, Moonsu Park, Hongji Ryu, Hyerin Yu, Sungsoo Park, Yoon‐Seok Roh, Key‐Hwan Lim, Jin Tae Hong, Sang‐Bae Han, Chun‐Woong Park, Seok‐Beom Yong, Hanseul Park

**Affiliations:** ^1^ Laboratory of Molecular Genetics College of Pharmacy Chungbuk National University Cheongju 28160 Republic of Korea; ^2^ Center for Gene & Cell Therapy Korea Research Institute of Bioscience and Biotechnology (KRIBB) Chungcheongbuk‐do 28116 Republic of Korea; ^3^ College of Veterinary Medicine Jeonbuk National University Iksan 54596 Republic of Korea; ^4^ College of Pharmacy Chungbuk National University Osongsaengmyeong 1‐ro, Osong‐eup, Heungdeok‐gu Cheongju 28160 Republic of Korea

**Keywords:** Alzheimer's disease, CRISPRa lipid nanocomplex, endocytosis, gene therapy, Mt3

## Abstract

Metallothionein 3 (Mt3) is crucial for cellular homeostasis and neuroprotection, with accumulating evidence linking it to amyloid‐beta (Aβ) clearance by astrocytes. This study developed a CRISPR activator (CRISPRa) system using lipid nanoparticles to selectively upregulate Mt3 in astrocytes, aiming to enhance Aβ endocytosis in an Alzheimer's disease (AD) mouse model. To directly assess the therapeutic potential of Mt3 activation in a specific brain region, stereotaxic injection is utilized to deliver the CRISPRa lipid nanocomplexes. This approach enabled precise in vivo brain delivery and Mt3 activation. The findings reveal that CRISPRa lipid nanocomplex‐mediated Mt3 upregulation significantly boosts Aβ uptake by astrocytes, leading to a marked reduction in Aβ plaque accumulation in AD mouse brains. These results highlight CRISPRa lipid nanocomplex‐mediated Mt3 targeting as a promising strategy to enhance endogenous Aβ clearance, presenting a novel therapeutic avenue for AD.

## Graphical abstract

GeTe exhibits intrinsically high carrier concentrations that limit its thermoelectric performance. Herein, CuAgSe alloying synergistically tunes the band structure, induces band flattening and valence‐band convergence, promotes Cu‐ion delocalization, and generates multiscale hierarchical structures to suppress phonon transport. This coordinated modulation of electrical and thermal properties synergistically enhances the thermoelectric performance of GeTe‐based materials.

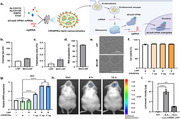

## 1 Introduction

Metallothionein‐3 (MT3) is a member of the metallothionein (MT) family, a group of low‐molecular‐weight, cysteine‐rich proteins found in all eukaryotic organisms.^[^ ^1^ ^]^ The MT family comprises four primary members, including MT1, MT2, MT3, and MT4, each with multiple isoform subclasses. While MT1 and MT2 are broadly expressed across various tissues, MT3 is predominantly localized in the central nervous system (CNS), and MT4 is confined to squamous epithelial tissues.^[^ ^2^ ^]^ Extensive research has revealed significant alterations in MT3 levels in the brains of Alzheimer's disease (AD) patients.^[^ ^2^, ^3^, ^4^ ^]^ Notably, MT3 expression is markedly reduced in AD, implicating its depletion in disease progression and heightened neuronal vulnerability.^[^ ^3^, ^4^, ^5^, ^6^, ^7^ ^]^ MT3 plays a key role in clathrin‐mediated endocytosis (CME) by binding to F‐actin and promoting its polymerization—an essential process for efficient CME.^[^ ^7^ ^]^ Additionally, MT3 regulates amyloid‐beta (Aβ) endocytosis in cortical astrocytes through its control of actin polymerization.^[^ ^7^ ^]^ Cytoskeletal dysfunction is a well‐established feature of neurodegenerative diseases such as AD, Parkinson's disease (PD), Amyotrophic lateral sclerosis, and Huntington's disease.^[^ ^8^ ^]^ Given MT3's influence on cytoskeletal dynamics, its role in neuroprotection is of particular interest.^[^ ^9^ ^]^ MT3 deficiency results in significant impairments in lysosomal biogenesis and cytoskeletal function,^[^ ^2^ ^]^ lysosomal dysfunction, and disrupted actin dynamics in MT3 knock‐out (KO) astrocytes, exacerbating neurodegeneration caused by toxic Aβ proteins and cellular debris.^[^ ^10^ ^]^ These findings position MT3 as a potential therapeutic target for neurodegenerative diseases. By regulating actin polymerization, which is crucial for cytoskeletal integrity and lysosomal function, MT3 may help mitigate disease progression by enhancing Aβ clearance and preserving neuronal health.

Regulating MT3 expression presents a promising avenue for AD treatment. Conventional gene therapy primarily employs cDNA‐based overexpression to enhance gene expression.^[^ ^11^ ^]^ However, this approach bypasses endogenous regulatory mechanisms and relies on strong exogenous promoters, often resulting in excessive protein production.^[^ ^11^ ^]^ Consequently, it may fail to replicate the physiological expression patterns necessary for maintaining cellular homeostasis.^[^ ^11^ ^]^ Clustered regularly interspaced short palindromic repeats (CRISPR)/Cas9‐mediated activation (CRISPRa) offers a precise and effective alternative for stimulating endogenous gene expression.^[^ ^12^ ^]^ The first‐generation CRISPRa system, dCas9‐VP64, was engineered by fusing the VP64 transcriptional activator to a catalytically dead Cas9 (dCas9).^[^ ^12^ ^]^ This modification enables dCas9 to enhance target gene expression by binding to promoters via guide RNA (gRNA).^[^ ^12^, ^13^ ^]^ Numerous studies have demonstrated the therapeutic applications of dCas9‐VP64 in gene therapy. For instance, a dual dCas9 transactivator system successfully reprogrammed astrocytes into GABAergic neurons in a PD model, leading to improved motor function.^[^ ^14^ ^]^ Another study utilized CRISPRa to regulate ITGB5, TIMP1, and TMEM176B in prostate cancer cells, demonstrating its potential to suppress cancer cell proliferation.^[^ ^15^ ^]^ These findings underscore the broad therapeutic potential of CRISPRa‐based strategies across multiple disease contexts. By enabling precise gene activation, CRISPRa offers a novel and targeted approach for modulating gene expression, presenting new possibilities for the treatment of neurodegenerative diseases and beyond.

For CRISPRa to be effectively utilized in AD therapy, a robust delivery system is essential for ensuring successful transport to the brain. However, achieving efficient delivery of CRISPRa components to the CNS remains a significant challenge.^[^ ^16^ ^]^ Among various delivery platforms, lipid nanoparticles (LNPs) have emerged as a leading nonviral system due to their high efficiency in nucleic acid transport.^[^ ^17^, ^18^ ^]^ Given the need for safe and effective genome editing, LNPs have also been explored as a promising vehicle for CRISPR/Cas9 delivery, offering low immunogenicity and high versatility.^[^ ^19^ ^]^ The success of ionizable LNP‐based mRNA vaccines for SARS‐CoV‐2 has further accelerated advancements in LNP technology for gene therapy applications.^[^ ^20^ ^]^ Recent progress in LNP formulations has enabled the targeted and efficient delivery of CRISPR‐based systems, including mRNA and ribonucleoprotein complexes.^[^ ^19^, ^21^ ^]^ This approach holds significant promise for overcoming delivery barriers and facilitating CRISPRa‐mediated gene regulation in AD treatment.

In this study, we developed a CRISPR lipid nanocomplex‐mediated gene activation system to selectively target and upregulate endogenous Mt3 expression in primary astrocytes and the brain, and evaluated its therapeutic potential by delivering the nanocomplexes into the brain via stereotaxic injection. Single‐cell RNA sequencing (scRNA‐seq) revealed that Mt3 expression was downregulated in 6‐month‐old AD model mice compared to 1‐month‐old controls. This reduction was effectively reversed using the CRISPRa lipid nanocomplex, restoring Mt3 expression both in primary astrocytes and in vivo. To evaluate the therapeutic potential of Mt3 activation, we examined Aβ42 plaque formation and Aβ‐associated memory impairment in the 5xFAD AD mouse model. Elevated Mt3 expression enhanced Aβ42 clearance through astrocytic endocytosis, highlighting its potential as a therapeutic target for AD. This study establishes Mt3‐targeted activation as a novel strategy for AD treatment.

## 2 Results

### 2.1 Downregulation of Mt3 in Astrocytes of AD Mouse Model and its Restoration via dCas9‐VP64‐Mediated Activation

**Figure 1:**
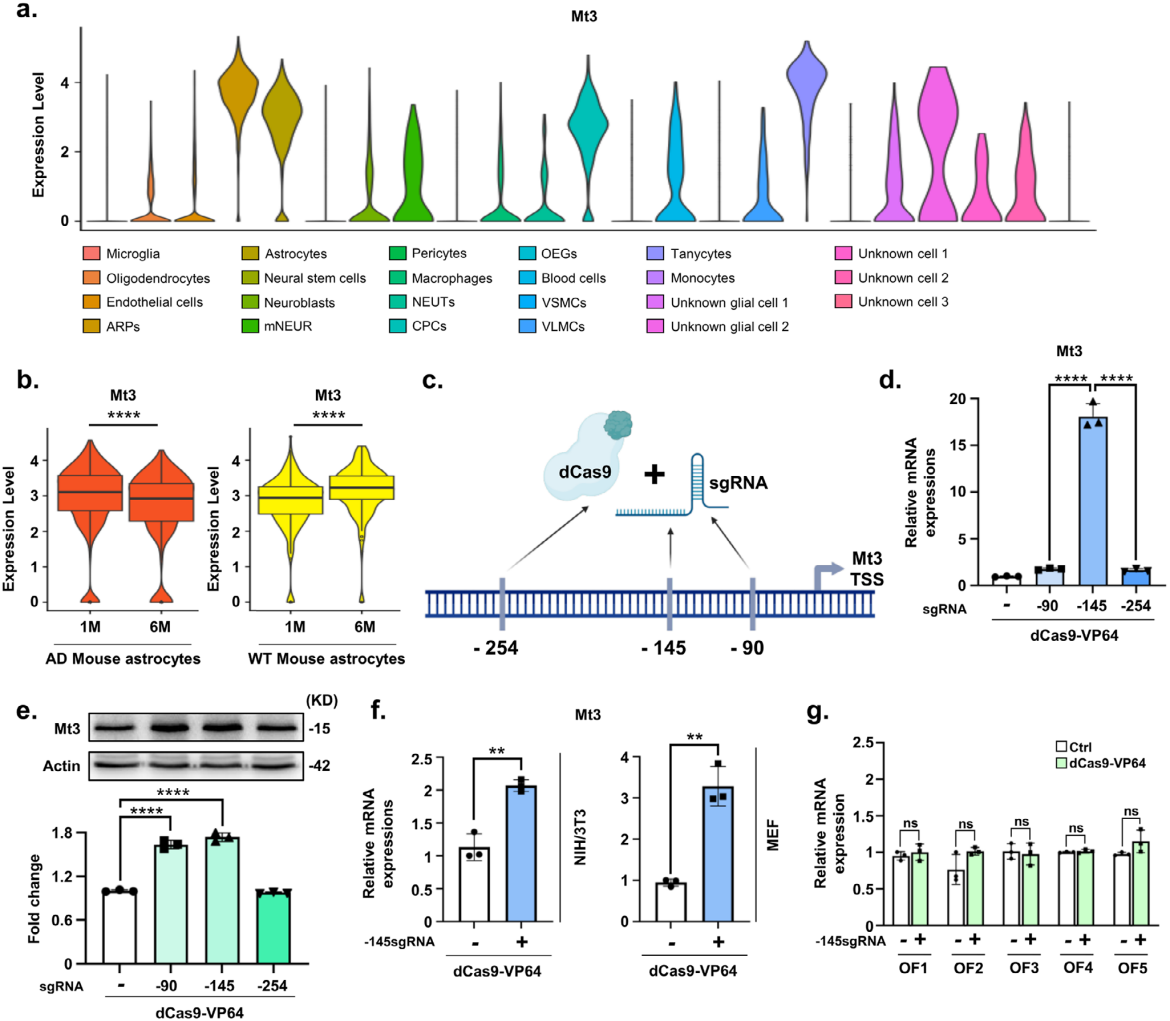
Downregulation of Mt3 in astrocytes of an AD mouse model and upregulation of Mt3 by the dCas9‐VP64 system. a) Violin plot illustrating the expression of Mt3 across different brain cell types in an AD mouse model. b) Comparison of Mt3 expression levels in astrocytes from WT and AD mouse models at 1 and 6 months of age. c) Schematic representation of the Mt3 promoter‐targeting sgRNAs designed for Mt3 allele activation (Created with BioRender). d) Quantitative real‐time PCR analysis of Mt3 mRNA expression in N2a cells treated with sgRNAs targeting −90, −145, and −254 bp upstream of the Mt3 gene start codon. Data are expressed as mean  ±  SEM (*n*  =  3). ^****^ *p*  <  0.0001, one‐way ANOVA with Tukey's multiple comparisons test. e) Western blot analysis of Mt3 protein levels in N2a cells treated with sgRNAs (−90, −145, or −254 bp). Data are expressed as mean  ±  SEM (*n*  =  3). ^****^ *p*  <  0.0001, one‐way ANOVA with Tukey's multiple comparisons test. f) qRT‐PCR analysis of Mt3 mRNA expression in NIH/3T3 cells and MEF cells treated with −145 bp sgRNA and dCas9‐VP64. Data are expressed as mean  ±  SEM (*n* =  3). ^**^ *p*  <  0.01, two‐sided Student's *t*‐test. g) qRT‐PCR analysis of the predicted off‐target genes in N2a cells treated with −145 bp sgRNA and dCas9‐VP64. Data are expressed as mean ± SEM (*n* = 3). ^*^ *p* < 0.05, two‐sided Student's *t*‐test. The image in e) is representative of three or more independent experiments. Cell type abbreviations: OEGs, Oligodendrocyte‐like Ependymal Glial cells; NEUTs, Neutrophils; VSMCs, Vascular Smooth Muscle Cells; ARPs, Astrocyte‐Related Precursors; mNEUR, Mature Neurons; CPCs, Choroid Plexus Cells; VLMCs, Vascular Leptomeningeal Cells.

Mt3 has gained significant attention due to its downregulation in the AD brain and its role in mitigating Aβ‐induced cytotoxicity.^[^ ^3^ ^,22]^ However, the specific cell populations affected by Mt3 depletion and the stages of AD in which this reduction occurs remain unclear. To investigate the relationship between Mt3 and AD, we conducted scRNA‐seq analysis. The scRNA‐seq data used in this study were derived from sequencing datasets previously published by our group.^[^ ^23^ ^]^ To identify Mt3‐associated cell types, we performed single‐cell transcriptome profiling using wild‐type (WT) and App knock‐in mice, which carry the Beyreuther/Iberian, Swedish, and Arctic mutations in the App gene locus.^[^ ^24^ ^]^ Clustering analysis identified 23 distinct cell clusters, annotated based on predefined marker genes (**Figure** **1**a). Further investigation revealed that Mt3 expressions were primarily enriched in astrocyte‐related clusters, including astrocyte‐related precursors (ARPs), astrocytes, and tanycytes (Figure 1a). A previous study demonstrated that Mt3 contributes to actin polymerization in astrocytes, playing a crucial role in supporting astrocytic endocytosis.^[^ ^7^ ^]^ Given this, we focused our analysis on the astrocyte cluster. We further examined Mt3 expressions in astrocytes across different stages of AD progression. The results showed a significant decline in Mt3 expression at 6 months in AD mice (Figure 1b). Interestingly, in age‐matched WT mice, Mt3 expression increased from 1 to 6 months of age, which is consistent with previous findings that Mt3 levels rise with normal aging in both rodents and humans (Figure 1b).^[^ ^25^, ^26^ ^]^ This age‐associated upregulation has been proposed to reflect a compensatory response to oxidative stress in the aging brain.^[^ ^27^ ^]^ Therefore, the Mt3 downregulation observed in AD mice likely reflects a disease‐specific pathological regulation, rather than a general age‐related or developmental change. These findings suggest that Mt3 expression is closely associated with astrocytes and that its levels decline as AD progression in the AD mouse model.

In this study, we investigated whether increased Mt3 expression in astrocytes could yield therapeutic effects in AD by regulating endogenous Mt3 gene expression via the dCas9‐VP64 system. We designed and tested single guide RNAs (sgRNAs) to activate endogenous Mt3 expression (Figure 1c; Table S1, Supporting Information). To determine the most effective binding sites for Mt3 induction, we selected three sgRNAs from the Mt3 promoter region and evaluated their transactivation efficiency in Neuro2a (N2a) cells. The results showed that N2a cells treated with both the sgRNA and dCas9‐VP64 exhibited significantly higher Mt3 mRNA expression than the control group (Figure 1d). Notably, transfection with the Mt3‐145 sgRNA, located 145 base pairs upstream of the ATG start codon, significantly increased Mt3 expression, yielding an activation fold approximately eighteen times greater than in the control group (Figure 1d; Figure S1a, Supporting Information). Protein level assessment further confirmed similar expression patterns following Mt3 activation (Figure 1e). Mt3 protein levels were notably higher in the −90 and −145 sgRNA‐targeted Mt3 groups (Figure 1e). Given these findings, we prioritized the −145 sgRNA for targeting Mt3 activation using the dCas9‐VP64 system. Mt3 activation was also validated in other cell types. We observed increased Mt3 expression in both mouse embryonic fibroblast (MEF) and NIH/3T3 cell lines following treatment with the −145 sgRNA and dCas9‐VP64 system (Figure 1f). Furthermore, applying CRISPR interference (CRISPRi) using the −145 sgRNA and dCas9‐KRAB system effectively inhibited Mt3 expression in both NIH/3T3 and MEF cells (Figure S1b, Supporting Information). To assess the specificity of the dCas9‐VP64 system, we examined potential off‐target effects in N2a cells. Using the Cas‐OFFinder web‐based tool, we identified and validated five predicted off‐target sites (Table S2, Supporting Information). Our analysis revealed no detectable changes at these sites following treatment with Mt3 sgRNA and dCas9‐VP64 (Figure 1g). These results indicate that the dCas9‐VP64 system selectively activates the Mt3 promoter, increasing Mt3 expression without any detectable off‐target effects.

### 2.2 Development and Characterization of CRISPRa Lipid Nanocomplex

To successfully apply CRISPR/Cas‐based gene therapy in vivo within the brain, selecting an effective delivery system is crucial. Although viral‐based delivery methods have been widely employed, they pose several challenges, including excessive immunogenicity and potential toxicity.^[^ ^28^ ^]^ LNPs have emerged as a clinically relevant, nonviral gene delivery platform, notably validated in mRNA vaccines for COVID‐19. They protect RNA cargo from enzymatic degradation and enable efficient intracellular delivery, making them well‐suited for mRNA therapeutics.^[^ ^29^ ^]^ Recent advances have demonstrated that LNPs can be engineered to traverse the blood‐brain barrier (BBB) and effectively deliver nucleic acids to various CNS‐resident cells, including neurons, astrocytes, and microglia.^[^ ^30^, ^31^ ^]^ These findings support their expanding utility in neurological disease models.^[^ ^30^, ^31^, ^32^ ^]^ Based on these properties, we aimed to deliver CRISRPa using LNPs.

**Figure 2:**
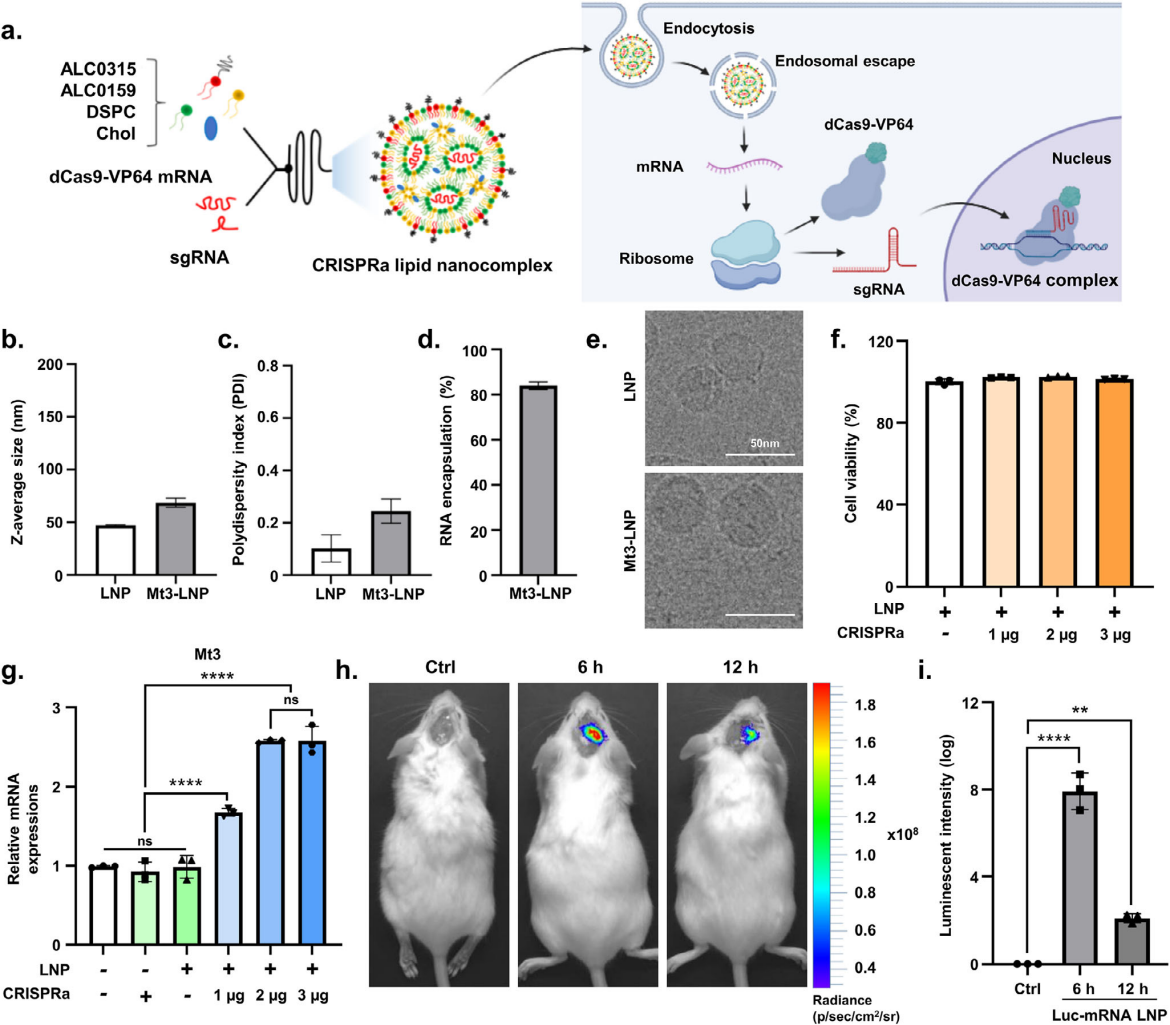
Characterization of CRISPRa lipid nanocomplex. a) Schematic illustration of Mt3 CRISPRa lipid nanocomplex preparation, showing microfluidic‐based lipid mixing for the construction of Mt3 CRISPRa lipid nanocomplexes encapsulating dCas9‐VP64 mRNA and sgRNA‐Mt3 (Created with BioRender). b,c) Physicochemical characterization of CRISPRa lipid nanocomplexes using dynamic light scattering assay. Data are presented as mean ± S.E.M., *n* = 6–12/group. d) RNA encapsulation efficiency of CRISPRa lipid nanocomplexes. Data are presented as mean ± S.E.M., *n* = 6–12/group. e) Cryo‐EM micrograph of CRISPRa lipid nanocomplexes, illustrating their structural characteristics. f) MTT cell viability assay of N2a cells treated with Empty‐LNP, or Mt3 CRISPRa lipid nanocomplexes at 1, 2, and 3 µg for 48 h post‐treatment. Data are expressed as mean  ±  SEM (*n*  =  3). ^*^ *p*  <  0.05, one‐way ANOVA with Tukey's multiple comparisons test. g) Quantitative real‐time PCR analysis of Mt3 gene expression in N2a cells treated with Empty‐LNPs, dCas9‐VP64 with Mt3 sgRNA or Mt3 CRISPRa lipid nanocomplexes at 1, 2, and 3 µg for 48 h post‐treatment. Data are expressed as mean  ±  SEM (*n*  =  3). ^*^ *p*  <  0.05, ^****^ *p*  <  0.0001, two‐way ANOVA with Tukey's multiple comparisons test. h) Luciferase expression following Lus‐mRNA LNP brain injection in ICR mice (*n*  =  3). Representative IVIS images of live animals and fluorescence signal quantification at the injection site. i) Quantification ratio of luciferase intensity from Figure 2h. Data are expressed as mean  ±  SEM (*n* =  3). ^**^ *p*  <  0.01, ^****^ *p*  <  0.0001, two‐way ANOVA with Tukey's multiple comparisons test. The images in (e,h) are representative of three or more independent experiments. Abbreviation: Mt3‐LNP, Mt3 CRISPRa lipid nanocomplex.

Herein, ALC0‐315‐based LNPs, the lipid components of Comirnaty (Pfizer's COVID‐19 mRNA vaccine), were employed for CRISPRa lipid nanocomplex preparation (**Figure** **2**a).^[^ ^20^, ^33^ ^]^ A weight ratio of 3:1 between CRISPRa mRNA and Mt3 sgRNA was chosen for LNP encapsulation, following a previous study.^[^ ^19^ ^]^ Dynamic light scattering (DLS) assay results showed that the CRISPRa lipid nanocomplex measured 68.37 nm, slightly larger than the 47.01 nm of RNA‐unloaded, empty‐LNPs (Figure 2b). Additionally, the CRISPRa lipid nanocomplex exhibited a higher PDI value compared to empty‐LNPs (0.24 vs 0.1; Figure 2c). The CRISPRa lipid nanocomplex also demonstrated RNA encapsulation efficiency exceeding 80% (Figure 2d). Cryo‐EM images revealed spherical morphologies for both LNPs, with the CRISPRa lipid nanocomplex appearing larger than the empty‐LNPs, a finding consistent with the DLS assay results (Figure 2e). Next, we evaluated the cytotoxicity of the CRISPRa lipid nanocomplex, which exhibited minimal cytotoxicity and had no impact on cell viability in vitro (Figure 2f). To assess the targeting efficiency of the CRISPRa lipid nanocomplex, CRISPRa lipid nanocomplexes were applied to N2a cells at varying concentrations, followed by qRT‐PCR analysis. The results showed that treatment with 3 µg of CRISPRa produced a similar increase in Mt3 RNA levels as 2 µg (Figure 2g). Therefore, 2 µg of CRISPRa was determined to be the most effective concentration for increasing Mt3 RNA levels and was selected for further in vitro experiments.

Although the BBB‐penetrating ability of ligand‐modified LNPs has been previously demonstrated,^[^ ^32^, ^34^ ^]^ the brain delivery efficiency of LNPs remains only 1%. Thus, stereotaxic injection presents a more practical delivery route for therapeutic applications. Accordingly, in this study, we aimed to deliver the CRISPRa lipid nanocomplex using the stereotaxic injection method. We assessed whether the CRISPRa lipid nanocomplex could effectively translate from in vitro to in vivo brain delivery. To do so, we stereotactically injected luciferase mRNA‐LNPs into the mouse brain. 6 and 12 h postinjection, luciferase activity was observed in the brain‐injected region (Figure 2h,i). In conclusion, the CRISPRa lipid nanocomplex was successfully formulated with efficient CRISPRa encapsulation. Targeting efficiency was validated in vitro, and stereotaxic injections demonstrated effective brain delivery, highlighting the potential of the CRISPRa lipid nanocomplex for in vivo applications.

### 2.3 CRISPRa Lipid Nanocomplex‐Mediated Activation of Mt3 Enhances Aβ Endocytosis in Astrocytes

**Figure 3:**
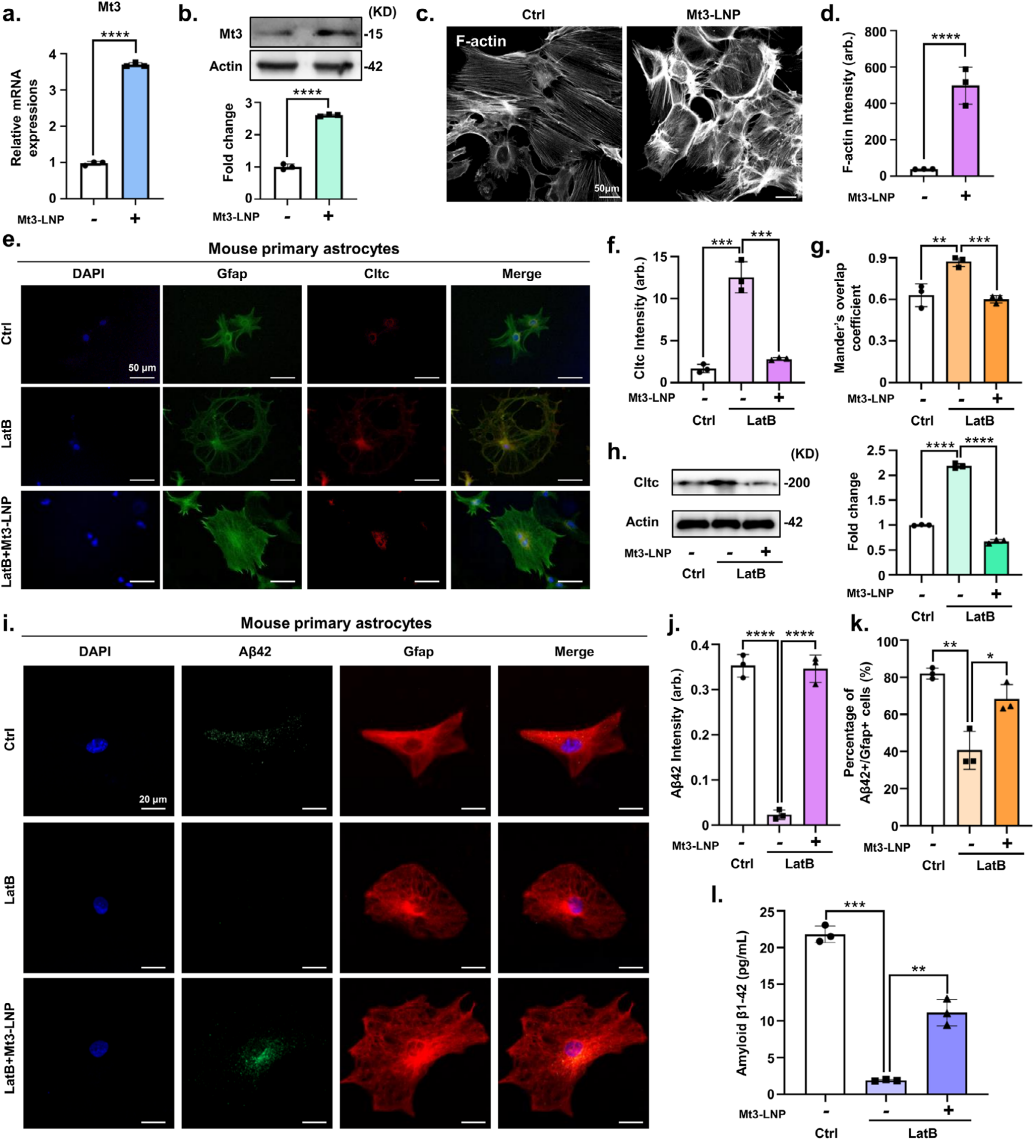
CRISPRa lipid nanocomplex‐mediated Mt3 activation enhances CME in primary mouse astrocytes. a) Quantitative real‐time PCR analysis of Mt3 gene expression in mouse primary astrocytes treated with Mt3 CRISPRa lipid nanocomplexes. Data are expressed as mean  ±  SEM (*n* =  3). ^****^ *p*  <  0.0001, two‐sided Student's *t*‐test. b) Western blot analysis of Mt3 protein levels in mouse primary astrocytes treated with Mt3 CRISPRa lipid nanocomplexes. Data are expressed as mean  ±  SEM (*n* =  3). ^****^ *p*  <  0.0001, two‐sided Student's *t*‐test. c) Immunostaining for Phalloidin‐iFluor 488 (F‐actin) in mouse primary astrocytes treated with Mt3 CRISPRa lipid nanocomplexes. d) Quantification ratio of F‐actin intensity from Figure 3c. Data are expressed as mean  ±  SEM (*n* =  3 biological replicates, with > 50 Gfap‐positive cells analyzed per group). ^****^ *p*  <  0.0001, two‐sided Student's *t*‐test. e) Immunostaining for Gfap (green), Cltc (red), and DAPI (blue) in mouse primary astrocytes treated with Mt3 CRISPRa lipid nanocomplexes. f) Quantification of Cltc intensity from Figure 3e. Data are expressed as mean  ±  SEM (*n* =  3). ^***^ *p*  <  0.001, two‐way ANOVA with Tukey's multiple comparisons test. g) Bar graph representing the degree of overlap between Cltc and Gfap signals, quantified using the MOC from Figure 3e. Data are expressed as mean  ±  SEM (*n* =  3). ^**^ *p*  <  0.01, ^***^ *p*  <  0.001, two‐way ANOVA with Tukey's multiple comparisons test. Over 100 astrocytes were analyzed for this experiment. h) Western blot analysis of Cltc protein levels in mouse primary astrocytes treated with Mt3 CRISPRa lipid nanocomplexes, followed by 1 µm LatB treatment for 1 h. Data are presented as mean ± SEM (*n* = 3). ^****^ *p* < 0.0001, two‐way ANOVA with Tukey's multiple comparisons test. i) Immunostaining of mouse primary astrocytes with Aβ42 (green), Gfap (red) and DAPI (blue) following a 2 day treatment with Mt3 CRISPRa lipid nanocomplexes, 1 h LatB treatment, and 15 min exposure to Aβ42 peptides. j) Quantification of Aβ42 intensity from Figure 3i. Data are expressed as mean  ±  SEM (*n* =  3 biological replicates, with > 50 Gfap‐positive cells analyzed per group). ^****^ *p*  <  0.0001, two‐way ANOVA with Tukey's multiple comparisons test. k) Percentage of Aβ42/Gfap double‐positive cells from Figure 3i. Data are expressed as mean  ±  SEM (*n* =  3 biological replicates, with > 50 Gfap‐positive cells per group). ^*^ *p*  <  0.05, ^**^ *p*  <  0.01, two‐way ANOVA Tukey's multiple comparisons test. Over 100 astrocytes were analyzed. l) ELISA measuring Aβ42 levels in mouse primary astrocytes. Data are expressed as mean  ±  SEM (*n* =  3). **p  <  0.01, ^***^ *p*  <  0.001, two‐way ANOVA with Tukey's multiple comparisons test. The images in (b,c,e,h, and i) are representative of three or more independent experiments.

Next, we hypothesized that activating Mt3 via the CRISPRa lipid nanocomplex could protect the brain from Aβ deposition by enhancing astrocytic endocytosis of Aβ, thereby reducing Aβ accumulation in neurons. Based on this, we reasoned that Mt3 activation would enhance astrocytic endocytosis, leading to reduced Aβ deposition. To evaluate the efficacy of the CRISPRa lipid nanocomplex for Mt3 gene activation, we conducted experiments in primary astrocytes derived from WT mice. Mt3 CRISPRa lipid nanocomplexes were delivered into WT mouse primary astrocytes, resulting in a significant increase in Mt3 expression at both RNA and protein levels, as assessed two days postdelivery, compared to the control (**Figure** **3**a,b). Endocytosis in cells primarily occurs through two mechanisms, clathrin‐dependent and caveolin‐dependent pathways.^[^ ^35^ ^]^ Previous studies have shown that Mt3 deletion impairs CME.^[^ ^7^ ^]^ Mt3 binds to F‐actin, enhancing actin polymerization, which is essential for efficient CME progression.^[^ ^36^ ^]^ Therefore, we investigated whether Mt3 activation via the CRISPRa lipid nanocomplex could enhance actin polymerization in astrocytes, thereby increasing CME. Overexpression of Mt3 using the CRISPRa lipid nanocomplex in astrocytes resulted in increased actin polymerization (Figure 3c,d). This finding aligns with previous studies demonstrating that Mt3 binds to F‐actin, leading to enhanced actin polymerization.^[^ ^36^ ^]^ To further confirm whether Mt3 alone is sufficient to influence clathrin‐dependent endocytosis, we used the actin‐disrupting drug latrunculin B (LatB), which disrupts actin polymerization, to assess the impact of Mt3 on CME in primary astrocytes. Following Mt3 upregulation via the CRISPRa lipid nanocomplex, we treated the cells with LatB and compared the results to a control group not treated with the CRISPRa lipid nanocomplex. Clathrin signaling exhibited an increased tendency in primary astrocytes treated with LatB compared to the untreated group (Figure 3e–g). However, Mt3 overexpression in primary astrocytes using the CRISPRa lipid nanocomplex partially reinstated the Mt3 control pattern, including clathrin localization and intensity (Figure 3e–g). Consistent with these findings, western blot analysis revealed that clathrin expression was higher in LatB‐treated cells compared to the control but returned to control levels upon Mt3 overexpression via the CRISPRa lipid nanocomplex (Figure 3h). Furthermore, to determine whether Mt3 activation affects caveolin‐mediated endocytosis, we examined both molecular and functional indicators of this pathway. The expression of Caveolin‐1 (Cav1), a key structural component of caveolae, was not altered by Mt3 activation via the CRISPRa lipid nanocomplex (Figure S2a, Supporting Information). To complement this result, we evaluated BSA‐488 uptake, which reflects caveolae‐mediated endocytosis. Primary astrocytes were incubated with BSA‐488 following Mt3 overexpression, and uptake was quantified by the proportion of BSA‐488/GFAP double‐positive cells. The percentage of BSA‐488/GFAP double‐positive astrocytes did not differ significantly between the control and Mt3‐overexpressing groups, further supporting the conclusion that Mt3 selectively enhances CME without affecting caveolin‐dependent pathways (Figure S2b–d, Supporting Information).

To further assess the specificity and potential off‐target effects of CRISPRa‐mediated Mt3 activation, we conducted in vitro validation studies in primary neurons and microglia (Figures S3 and S4, Supporting Information). In primary neurons, Mt3 expression was significantly upregulated following CRISPRa lipid nanocomplex treatment, without affecting cell viability (Figure S3a,b). Immunostaining with Tuj1 and NeuN revealed that Mt3 activation did not alter neuronal number or neurite lengths (Figure S3c–e, Supporting Information), suggesting that Mt3 overexpression does not adversely affect neuronal phenotype. In primary microglia, CRISPRa lipid nanocomplexes similarly increased Mt3 expression without reducing cell viability (Figure S4a,b, Supporting Information). Notably, in an inflammatory context induced by lipopolysaccharide (LPS) stimulation, Mt3 activation led to decreased expression of pro‐inflammatory cytokines TNF‐α, IL‐1β, and IL‐6 (Figure S4c, Supporting Information). Moreover, immunostaining for CD206 and CD16/32 demonstrated a shift toward an M2‐like anti‐inflammatory phenotype in Mt3‐overexpressing microglia (Figure S4d–f, Supporting Information). This observation is consistent with prior studies in macrophages, where Mt3 supported M2 polarization,^[^ ^37^ ^]^ suggesting that similar immunomodulatory effects may also occur in microglia. These results indicate that Mt3 activation in non‐astrocytic brain cells is not only safe but may also provide auxiliary therapeutic benefits by modulating neuroinflammation.

Next, we investigated the intracellular endocytic pathways involved in Aβ uptake by astrocytes. Astrocytes were treated with Mt3 CRISPRa lipid nanocomplexes for two days, followed by a 1 h treatment with LatB. Aβ peptides were then added for 15 min to assess Aβ uptake. The presence of the Aβ42 peptide within astrocyte cell bodies confirmed their endocytic function (Figure 3h–k). Furthermore, LatB treatment significantly reduced the number of Aβ42/GFAP double‐positive cells (Figure 3i–k). In contrast, Mt3 overexpression increased the number of Aβ42/GFAP double‐positive cells (Figure 3i–k). Additionally, Enzyme‐linked immunosorbent assay (ELISA) analysis confirmed that intracellular Aβ42 levels in astrocytes further increased when Mt3 was upregulated in the LatB‐treated group (Figure 3l). Consistently, when astrocytes were treated with Aβ oligomers, Mt3 overexpression also increased Aβ42 intensity and the percentage of Aβ42/Gfap double‐positive cells, even in the presence of LatB (Figure S5a–c, Supporting Information). These results demonstrate that Mt3 overexpression via CRISPRa lipid nanocomplexes enhances actin polymerization, thereby promoting the endocytosis of Aβ42.

### 2.4 CRISPRa Lipid Nanocomplex‐Mediated Activation of Mt3 in Astrocytes Reduces Aβ42 Accumulation in Primary Neurons

**Figure 4:**
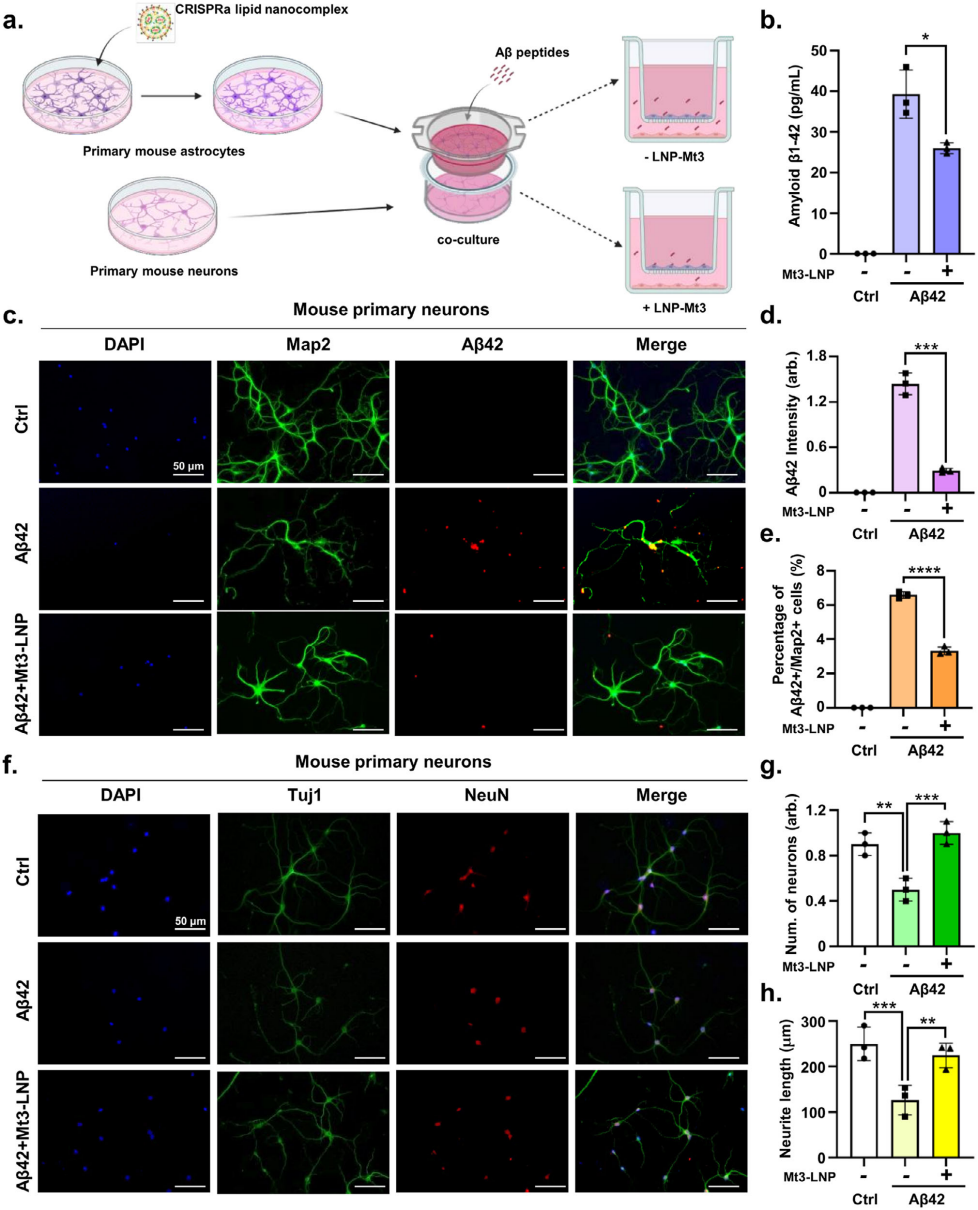
CRISPRa lipid nanocomplex‐mediated Mt3 activation in astrocytes reduces Aβ42 accumulation in cocultured primary mouse neurons. a) Schematic representation of the primary astrocyte and primary neuron coculture system. Astrocytes are first treated with CRISPRa lipid nanocomplexes for two days, followed by coculture with primary neurons. During the coculture period, cells are exposed to Aβ42 peptides for subsequent analysis (Created with BioRender). b) ELISA measuring Aβ42 levels in mouse primary neurons. Data are expressed as mean  ±  SEM (*n* =  3). ^*^ *p*  <  0.05, two‐way ANOVA with Tukey's multiple comparisons test. c) Immunostaining for Map2 (green), Aβ42 (red), and DAPI (blue) in mouse primary neurons. d) Quantification of Aβ42 intensity from Figure 4c. Data are expressed as mean  ±  SEM (*n* =  3). ^***^ *p*  <  0.001, two‐way ANOVA with Tukey's multiple comparisons test. e) Percentage of Aβ42/Map2 double‐positive cells from Figure 4c. Data are expressed as mean  ±  SEM (*n* =  3). ^****^ *p*  <  0.0001, two‐way ANOVA with Tukey's multiple comparisons test. Over 100 Map2‐positive neurons were analyzed. f) Immunostaining for Tuj1 (green), NeuN (red), and DAPI (blue) in mouse primary neurons. g) Quantification of Tuj1‐positive neurons from Figure 4f. Data are expressed as mean  ±  SEM (*n* =  3). ^**^ *p*  <  0.01, ^***^ *p*  <  0.001, two‐way ANOVA with Tukey's multiple comparisons test. h) Quantification of neurite length in primary neurons. Data are expressed as mean  ±  SEM (*n* =  3). ^**^ *p*  <  0.01, ^***^ *p*  <  0.001, two‐way ANOVA with Tukey's multiple comparisons test. Over 100 neurites were measured. The images in (c,f) are representative of three or more independent experiments.

Mt3 overexpression via CRISPRa lipid nanocomplexes also enhances Aβ42 endocytosis in astrocytes, potentially reducing Aβ42 levels in neurons. To investigate this, we co‐cultured primary neurons and astrocytes. Primary astrocytes were treated with Mt3 CRISPRa lipid nanocomplexes for 48 h. To examine whether astrocyte‐mediated endocytosis directly affects neurons, we isolated primary neurons from mouse embryonic brains at 14.5 dpc and co‐cultured them with either CRISPRa lipid nanocomplex‐treated or untreated astrocytes. Aβ42 peptides were then added to the co‐cultured cells, and primary neurons were evaluated (**Figure** **4**a). First, we assessed Aβ42 levels in neurons. ELISA analysis revealed a significant reduction in Aβ42 levels in neurons co‐cultured with Mt3 CRISPRa lipid nanocomplex‐treated astrocytes (Figure 4b). Furthermore, the number of Aβ42/Map2 double‐positive neurons decreased in neurons co‐cultured with Mt3 CRISPRa lipid nanocomplex‐treated astrocytes (Figure 4c–e). Next, we assessed neuron morphology in relation to Aβ42 reduction. A decrease in neuron number and neurite length was observed in primary neurons treated with Aβ42 peptides. However, delivery of Mt3 CRISPRa lipid nanocomplexes increased neurite length and neuron number (Figure 4f–h). These findings demonstrate that Mt3 activation in astrocytes prevents Aβ‐induced AD pathology associated with neurodegeneration.

### 2.5 In Vivo CRISPRa Lipid Nanocomplex‐Mediated Mt3 Activation in Mouse Brain

**Figure 5:**
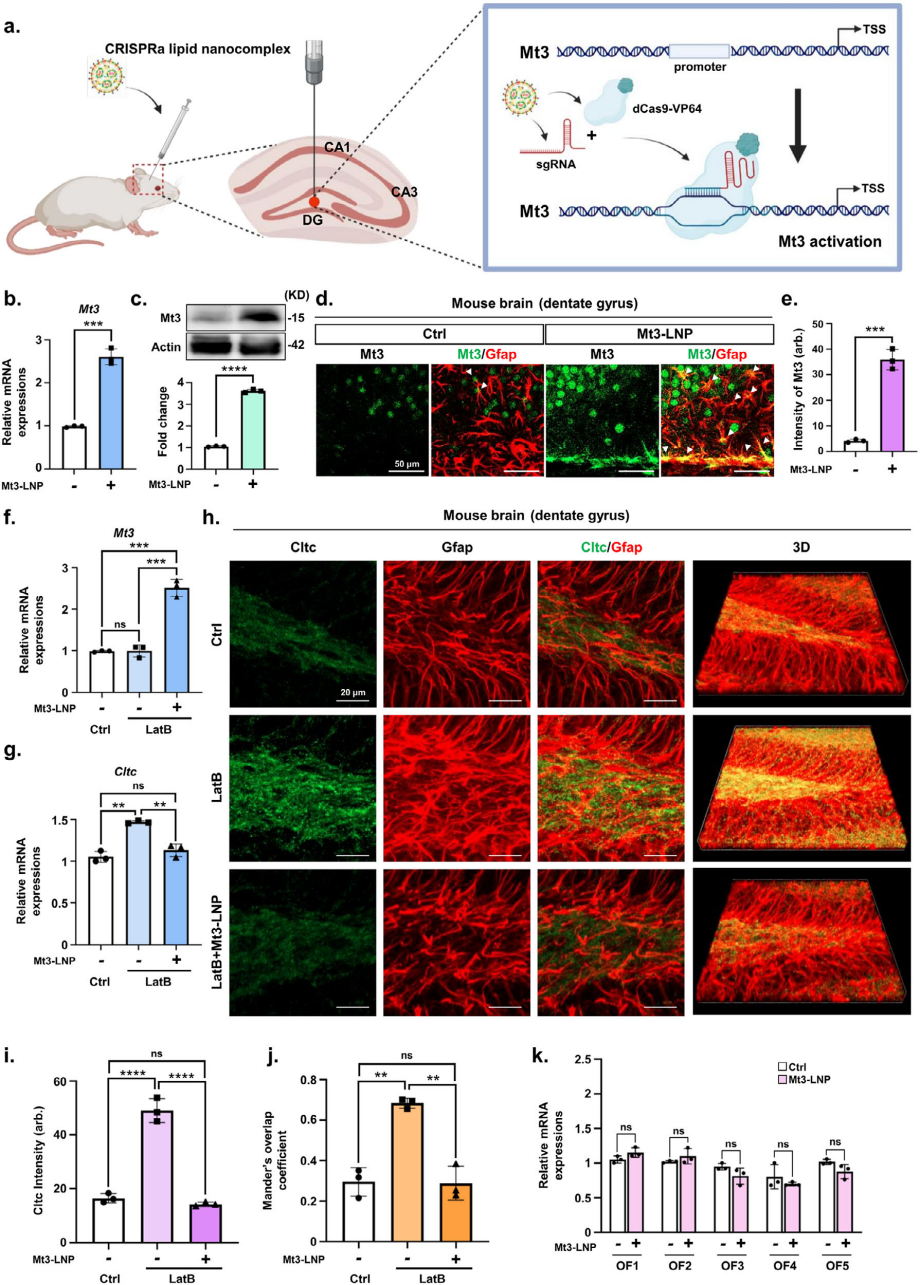
CRISPRa lipid nanocomplex‐mediated Mt3 activation in WT mouse brain in vivo. a) Schematic representation of Mt3 CRISPRa lipid nanocomplex‐mediated activation targeting Mt3 in the DG of the mouse brain (Created with BioRender). b) Quantitative real‐time PCR analysis of Mt3 expression in the WT mouse brain injected with or without Mt3 CRISPRa lipid nanocomplex. Data are expressed as mean  ±  SEM (*n* =  3). ^***^ *p*  <  0.001, two‐sided Student's *t*‐test. c) Western blot analysis of Mt3 expression in the WT mouse brain injected with Mt3 CRISPRa lipid nanocomplex. Data are expressed as mean ± SEM (*n* =  3). ^****^ *p* <  0.0001, two‐sided Student's *t*‐test. d) Immunostaining for Mt3 (green) and Gfap (red) in WT mouse brain injected with Mt3 CRISPRa lipid nanocomplexes. Arrowheads indicate regions with strong colocalization of Gfap and Mt3 signals. e) Quantification of Mt3 intensity from Figure 5d. Data are expressed as mean  ±  SEM (*n* =  3). ^***^ *p*  <  0.001, two‐sided Student's *t*‐test. f) qRT‐PCR analysis of Mt3 expression in the mouse brain after injection of 500 µm LatB. Mice were either treated or untreated with Mt3 CRISPRa lipid nanocomplex. Data are expressed as mean  ±  SEM (*n* =  3). ^*^ *p*  <  0.05, ^***^ *p*  <  0.001, two‐way ANOVA with Tukey's multiple comparisons test. g) qRT‐PCR analysis of Cltc expression in the WT mouse brain injected with 500 µm LatB and Mt3 CRISPRa lipid nanocomplex. Data are expressed as mean  ±  SEM (*n* =  3). ^*^ *p*  <  0.05, ^**^ *p*  <  0.01, two‐way ANOVA with Tukey's multiple comparisons test. h) Immunostaining for Cltc (green), Gfap (red), and DAPI (blue) after injecting Mt3 CRISPRa lipid nanocomplex directly into the DG of the WT mouse brain. 3D images were added to help visualize regions where Gfap and Cltc signals strongly colocalize (3D). i) Quantification ratio of Cltc intensity from Figure 5h. Data are expressed as mean  ±  SEM (*n* =  3). ^*^ *p*  <  0.05, ^****^ *p*  <  0.0001, two‐way ANOVA with Tukey's multiple comparisons test. j) Bar graph representing the degree of overlap between Cltc and Gfap signals, quantified using MOC from Figure 5h. Data are expressed as mean  ±  SEM (*n* =  3). ^*^ *p*  <  0.05, ^**^ *p*  <  0.01, two‐way ANOVA with Tukey's multiple comparisons test. Over 100 astrocytes were analyzed. k) qRT‐PCR analysis of predicted off‐target genes in the WT mouse brain injected with Mt3 CRISPRa lipid nanocomplex. Data are expressed as mean ± SEM (*n* = 3). ^*^ *p*  <  0.05, two‐sided Student's *t*‐test. The images in (c,d,h) are representative of three or more independent experiments.

To assess whether CRISPRa lipid nanocomplex‐mediated gene activation could facilitate in vivo Mt3 activation, we injected Mt3 CRISPRa lipid nanocomplexes directly into the dentate gyrus (DG) of the mouse brain (**Figure** **5**a). The DG is one of the earliest and most vulnerable hippocampal regions affected by AD pathology.^[^ ^38^ ^]^ Moreover, substantial Aβ plaque accumulation has been reported in this area,^[^ ^39^ ^]^ and astrocytes in the DG are actively involved in Aβ clearance through endocytosis and autophagy.^[^ ^40^ ^]^ Based on these pathological and functional characteristics, we selected the DG as the injection site to maximize the therapeutic impact of Mt3 activation. Delivery of Mt3 CRISPRa lipid nanocomplexes significantly increased both mRNA expression and protein levels of Mt3 in the mouse brain (Figure 5b–e). Next, to determine whether the Mt3 CRISPRa lipid nanocomplex influenced actin polymerization, we assessed actin dynamics in the brain following treatment with Mt3 CRISPRa lipid nanocomplexes. After increasing Mt3 expression via CRISPRa lipid nanocomplexes, we treated the brains with LatB, an actin‐disrupting drug, and compared the results with a control group that did not receive CRISPRa lipid nanocomplex treatment (Figure 5f–j). In the LatB‐treated group, administration of CRISPRa lipid nanocomplexes successfully increased Mt3 expression in the brain (Figure 5f). Furthermore, clathrin signaling exhibited an increasing trend in LatB‐treated brains, where actin polymerization was inhibited (Figure 5g–j). However, Mt3 overexpression via CRISPRa lipid nanocomplexes partially restored clathrin intensity (Figure 5g–j). To further assess potential in vivo off‐target effects of the CRISPRa lipid nanocomplex in the brain, predicted off‐target sites were analyzed (Figure 5k). The results confirmed that the Mt3 CRISPRa lipid nanocomplex exhibited no off‐target effects (Figure 5k). These findings demonstrate that the CRISPRa lipid nanocomplex enables efficient gene activation in the brain, minimizes off‐target effects, and promotes actin polymerization.

### 2.6 Activation of Mt3 via CRISPRa Lipid Nanocomplex Reduces Aβ42 and Improves Cognition in the AD Mouse Model

**Figure 6:**
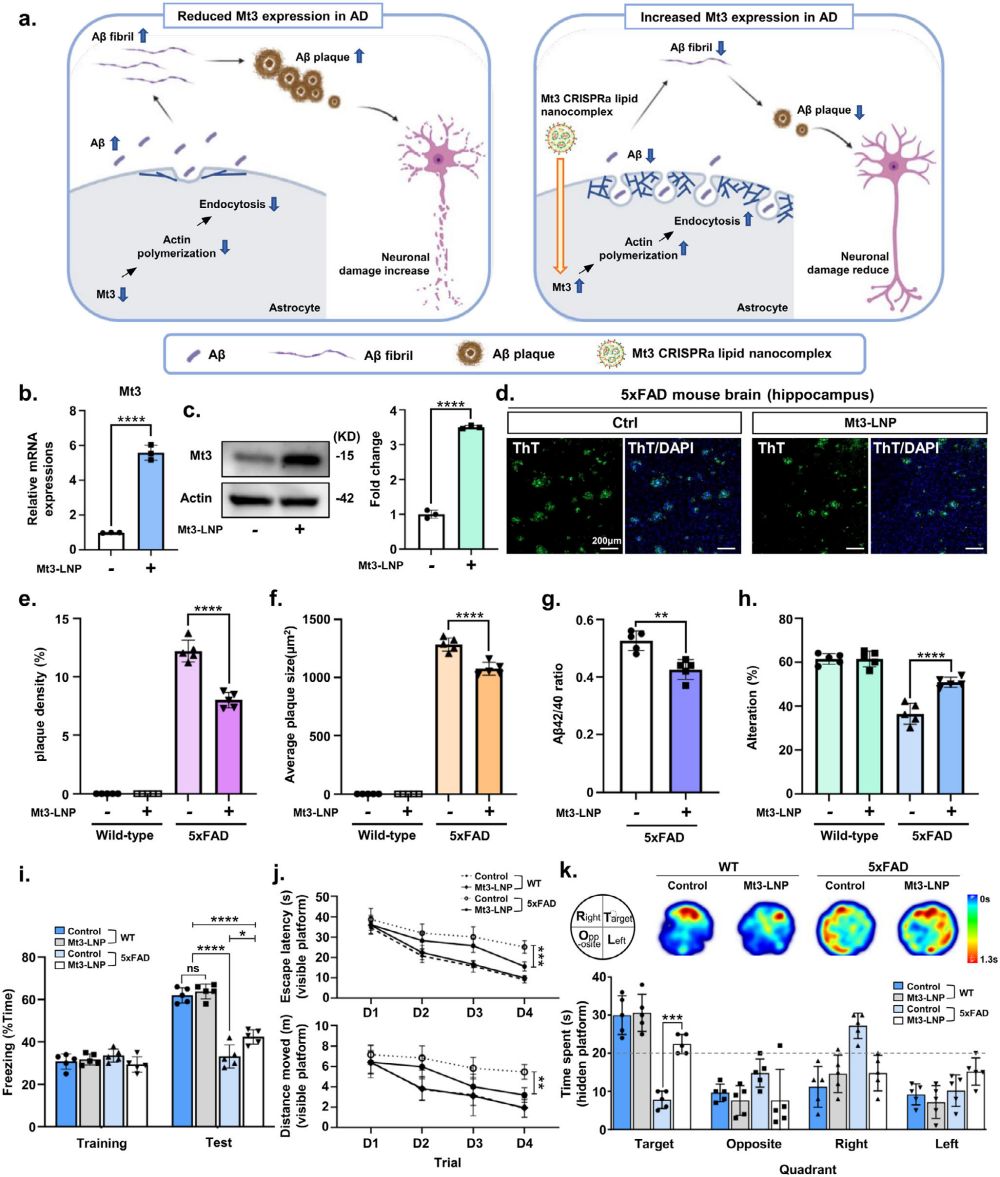
Mt3 CRISPRa lipid nanocomplex rescues Aβ accumulation and cognitive function in an AD mouse model. a) Schematic representation of Mt3 CRISPRa lipid nanocomplex‐mediated activation to enhance Aβ endocytosis, thereby reducing Aβ accumulation and mitigating cognitive deficits in the 5xFAD AD mouse model (Created with BioRender). b) Quantitative real‐time PCR analysis of Mt3 expression in the 5xFAD mouse brain injected with Mt3 CRISPRa lipid nanocomplex. Data are expressed as mean  ±  SEM (*n* =  3). ^****^ *p*  <  0.0001, two‐sided Student's *t*‐test. c) Western blot analysis of Mt3 expression in the 5xFAD mouse brain injected with Mt3 CRISPRa lipid nanocomplex. Data are expressed as mean  ±  SEM (*n* =  3). ^****^ *p* <  0.0001, two‐sided Student's *t*‐test. d) ThT assay for Aβ aggregation. ThT (green) and DAPI (blue) immunostaining in the brain of 5xFAD mice injected with Mt3 CRISPRa lipid nanocomplex. e,f) Quantification of Aβ42 deposition from Figure 6d, including plaque density and average plaque size. Data are shown as mean ± SEM (*n* = 5). ^****^ *p* < 0.0001, two‐way ANOVA with Tukey's multiple comparisons test. g) ELISA analysis of the Aβ42/Aβ40 ratio in the brains of 5xFAD mice injected with Mt3 CRISPRa lipid nanocomplex. Data are presented as mean ± SEM (*n* = 5). ^**^ *p* < 0.01, two‐sided Student's *t*‐test. h) Spontaneous alternation in the Y‐maze for wild‐type (WT) and 5xFAD mice injected with Mt3 CRISPRa lipid nanocomplex. Data are expressed as mean ± SEM. ^****^ *p* < 0.0001, two‐way ANOVA with Tukey's multiple comparisons test. i) Percentage of freezing response observed during the contextual fear memory test. Data are presented as mean ± SEM. ^****^ *p <* 0.0001, ^*^ *p <* 0.05, two‐way ANOVA with Tukey's multiple comparisons test. j,k) Long‐term spatial memory assessment using the Morris water maze. (j) Results of the visual platform training sessions, with the top graph showing escape latency and the bottom graph showing total distance traveled. Data are expressed as mean ± SEM. ^**^ *p* < 0.01, ^***^ *p* < 0.001, two‐way ANOVA with Tukey's multiple comparisons test. (k) Hidden platform test results, including a heat map and quantification of time spent in each quadrant. Data are presented as mean ± SEM. ^***^ *p* < 0.001, two‐way ANOVA with Tukey's multiple comparisons test. The images in panels (c,d) are representative of three or more independent experiments.

Previous studies have shown that in Mt3 KO mice, lysosomal dysfunction and disrupted actin dynamics exacerbate neurodegeneration triggered by toxic Aβ protein.^[^ ^7^ ^]^ Based on this, we investigated whether Mt3 activation in the brain via CRISPRa lipid nanocomplexes could reduce Aβ levels and mitigate memory impairments in an AD mouse model. Mt3‐targeting CRISPRa lipid nanocomplexes were injected into the DG region of 5‐month‐old 5xFAD AD model mice. Four weeks post‐delivery, the AD model mice underwent both biochemical and behavioral assessments (**Figure** **6**a). To evaluate in vivo delivery of the CRISPRa lipid nanocomplex, we examined Mt3 expression in the brain and observed a significant increase following CRISPRa lipid nanocomplex delivery (Figure 6b,c). Next, to gain a comprehensive understanding of the effects of Mt3 activation on Aβ pathology via CRISPRa lipid nanocomplexes, we assessed its impact on Aβ formation and memory impairment. Notably, CRISPRa lipid nanocomplex delivery significantly reduced ThT‐positive Aβ plaque accumulation in the hippocampus of AD mice compared to age‐matched AD model controls (Figure 6d–f). We also validated the decrease in Aβ40, Aβ42, and the Aβ42/Aβ40 ratio in CRISPRa lipid nanocomplex‐treated 5xFAD mouse brains (Figure 6g; Figure S6a,b, Supporting Information). These findings suggest that Mt3 activation via CRISPRa lipid nanocomplexes effectively reduces Aβ levels in AD mice. This Aβ accumulation reduction may result from enhanced astrocytic endocytosis facilitated by Mt3 activation,^[^ ^7^ ^]^ highlighting the potential of this targeted approach in alleviating Aβ pathology in AD models.

One of the main features of AD is cognitive decline, primarily due to neuronal loss caused by the progressive accumulation of Aβ42.^[^ ^41^ ^]^ To determine whether the CRISPRa lipid nanocomplex targeting Mt3 could influence cognition‐related behaviors, we conducted behavioral assessments on 5xFAD mice. Spatial and procedural working memory was evaluated using the Y‐maze test, where AD mice initially exhibited a lower alternation rate. However, the CRISPRa lipid nanocomplex‐treated group showed a significant improvement in the alternation rate compared to untreated age‐matched AD controls (Figure 6h). Short‐term reference memory was further assessed through the contextual fear conditioning test, which involves associative memory tasks dependent on the hippocampus. Interestingly, CRISPRa lipid nanocomplex‐treated AD mice exhibited a significant increase in freezing behavior, indicating improved memory retention (Figure 6i). Long‐term spatial memory was evaluated using the Morris water maze test by measuring time spent in each quadrant after a 4 day training phase. In trials with a visible platform, escape latency and distance showed significant differences across groups on day 4 (Figure 6j). However, when the platform was removed, CRISPRa lipid nanocomplex‐treated AD mice spent noticeably more time in the target quadrant than controls, suggesting better spatial memory retention (Figure 6k). Collectively, these findings suggest that CRISPRa lipid nanocomplex treatment may preserve learning capacity and enhance memory retention in AD mice. Taken together, this approach holds promise as a novel treatment strategy for AD and potentially other neurodegenerative diseases.

## 3 Discussion

Here, we demonstrated that CRISPRa lipid nanocomplex‐mediated gene activation of Mt3 in astrocytes can be applied therapeutically in an AD mouse model. The CRISPRa lipid nanocomplexes efficiently upregulated Mt3 expression in vivo brain, primarily in astrocytes. This upregulation facilitated astrocytic Aβ endocytosis in the AD mouse model, leading to a significant reduction in Aβ plaque accumulation. Consequently, the decreased Aβ accumulation helped mitigate neurotoxic effects, ultimately improving cognitive function and behavioral performance. Although astrocytes were the primary target of the CRISPRa system, Mt3 upregulation was also detected in neurons and microglia. To further evaluate cell‐type‐specific effects, we conducted in vitro validation experiments, which revealed M2‐like anti‐inflammatory polarization in Mt3‐overexpressing microglia, while Mt3 activation in neurons did not induce detectable toxicity or phenotypic alterations. These findings suggest that enhancing Mt3 expression in astrocytes directly contributes to Aβ clearance and may help reverse AD‐related pathology. Moreover, the additional immunomodulatory role of Mt3 in microglia may further amplify therapeutic benefits, warranting further studies to elucidate its underlying mechanisms.

In this study, the therapeutic effects observed through Mt3 activation were made possible by the application of an efficient and clinically viable gene delivery platform. CRISPRa lipid nanocomplexes, offer advantages over traditional viral vectors, which are limited by immunogenicity and cargo capacity.^[^ ^42^ ^]^ LNPs, in particular, represent a clinically validated delivery platform with high biocompatibility, biodegradability, and the ability to protect and transport nucleic acids.^[^ ^43^ ^]^ LNPs have already enabled the clinical translation of siRNA drugs and mRNA‐based COVID‐19 vaccines, demonstrating safety and efficacy in large‐scale human applications.^[^ ^44^, ^45^ ^]^ Furthermore, LNPs can be engineered for improved stability, reduced immune activation, and targeted tissue delivery—features that are critical for the success of in vivo gene therapies such as CRISPRa. Therefore, the CRISPRa lipid nanocomplex approach developed in this study holds strong potential as a safe and effective in vivo gene therapy strategy for AD.

Despite these promising results, several challenges must be addressed before CRISPRa lipid nanocomplex‐based therapies can be clinically applied to AD patients. One critical aspect is achieving astrocyte‐specific targeting to maximize therapeutic efficacy while minimizing off‐target effects. Developing antibody‐conjugated LNPs that selectively bind to astrocytes could enhance delivery precision, ensuring effective gene activation with minimal systemic toxicity. Additionally, determining the optimal timing for CRISPRa lipid nanocomplex administration is essential for improving disease outcomes. In this study, the complex was delivered after Aβ42 accumulation and plaque formation. However, earlier intervention, administering treatment at the onset of Aβ42 production, could potentially prevent plaque formation, leading to enhanced therapeutic benefits. Moreover, evaluating the long‐term effects of CRISPRa‐mediated Mt3 activation is necessary to determine the durability of Aβ clearance. Future studies should assess how long a single injection remains effective, with follow‐up observations at 3–6 months to evaluate sustained Aβ reduction and overall disease progression. Lastly, enhancing the brain delivery efficiency of LNPs is a critical area for further development. This method was used solely as a proof‐of‐concept experimental approach to directly evaluate the therapeutic potential of Mt3 activation in a localized brain region, rather than as a clinically practical delivery strategy. Because systemic administration typically results in less than 1% brain accumulation due to the restrictive nature of the BBB, we employed stereotaxic injection in this study. However, future approaches could improve LNP design through strategies such as ligand conjugation for receptor‐mediated transcytosis and incorporation of ApoE‐mimetic peptides. These improvements may enable efficient and non‐invasive CNS delivery for broader therapeutic applicability.

## 4 Conclusion

Taken together, these findings underscore the potential of CRISPRa lipid nanocomplex‐mediated Mt3 activation as a novel therapeutic strategy for AD. Further optimization of delivery specificity, timing, and long‐term efficacy will be essential to advancing this approach toward clinical application.

## 5 Experimental Section

### Ethics Statement

All experimental procedures and care of animals in this study were carried out according to the Institutional Animal Care and Use Committee at Chungbuk National University (CBNUA‐25‐0018‐02).

### Production of Guide RNAs

To clone a single guide RNA (sgRNA), we used the pAC152‐dual‐dCas9VP64‐sgExpression vector (Addgene, 48238). The vector was linearized with the BsmbI enzyme (Enzynomics) at 56 °C for 2 h. After linearization, the sgRNA oligonucleotide pairs were annealed with T4 polynucleotide kinase (NEB) and ligated into the linearized vector (50 ng) using T4 DNA ligase (NEB) for 2 h at room‐temperature. The ligation product was transformed into DH5α competent E. coli cells (Enzynomics) and the ligated vector was sequenced for confirmation (hU6 primer: GAG GGC CTA TTT CCC ATG ATT).

### In Vitro Transcription

Double‐stranded DNA templates generated by PCR were used for in vitro transcription to synthesize sgRNAs using the MEGAshortscript Transcription Kit (Thermo Fisher Scientific) according to the manufacturer's instructions. Double‐stranded DNA templates generated by PCR were used for in vitro transcription to synthesize dCas9‐VP64 mRNAs using the mMESSAGE mMACHINE T7 ULTRA Transcription Kit (Invitrogen) according to the manufacturer's instructions. The resulting sgRNAs and dCas9‐VP64 mRNAs were precipitated with ethanol and reconstituted in nuclease‐free water.

### CRISPRa Lipid Nanocomplex Preparation

Ionizable lipid (ALC0315) and PEG‐lipid (ALC0159) were purchased from BroadPharm (San Diego, CA, USA). Cholesterol and DSPC were purchased from Avanti Polar Lipids Inc. Empty‐ and CRISPRa‐LNPs were prepared as described in a previous study.^[^ ^17^, ^18^ ^]^ Briefly, one volume of lipids (Cholesterol, ALC0315, DSPC, ALC0159 at 45:43.5:10:1.5 molar ratio in EtOH) were mixed with three volumes of mRNA (dCas9‐VP64 mRNA:Mt3 sgRNA at 3:1 weight ratio in a sodium acetate buffer(pH4.5)) through a microfluidic device Nanoassemblr, IGNITE (Precision Nanosystems Inc) at a flow rate of 12 mL min^−1^. The prepared CRISPRa lipid nanocomplexes were dialyzed against phosphate‐buffered saline (PBS, pH 7.4) for 16 h.

### Quantification of RNA Encapsulation

The Quant‐iT RiboGreen RNA assay kit (Thermo Fisher Scientific) was used to evaluate the RNA encapsulation efficiency of LNPs. In brief, 1 µL of LNP was diluted in a final volume of 200 µL of TE buffer (20 mm EDTA, 10 mm Tris‐HCL) in the presence or absence of Triton X‐100 (0.5%, Sigma). Samples were loaded on a 96‐well black plate (SPL) and the plate was incubated for 15 min at 37 °C before adding 100 µL of reagent‐containing TE buffer (0.5% v/v, RiboGreen reagent) to each well. The fluorescence intensity was measured using a Tecan microplate reader (TECAN) according to the manufacturer's protocol.

### Dynamic Light Scattering (DLS) Analysis

Nano‐size and poly dispersity index of CRISPRa lipid nanocomplexes were measured by dynamic light scattering (DLS) using a Malvern nano ZS ζ‐sizer (Malvern Instruments). Briefly, PBS‐dialyzed LNP samples were diluted in distilled water (1:30, volume ratio) for DLS measurement.

### Cryo‐EM Imaging

To prepare cryo‐EM grids, empty‐LNPs or CRISPRa lipid nanocomplexes (4 µL) were applied to Quantifoil holey carbon EM grids (R1.2/1.3, 300 mesh; EMS). The EM grid was glow‐discharged for 90 s at 15 mA before sample application. Grid was blotted with Vitrobot Mark IV (FEI) using 3 s blotting time with 100% relative humidity at 4 °C. Samples were imaged on Glacios (FEI) at an acceleration voltage of 200 kV with Falcon IV direct electron detector (FEI). Cryo‐EM images were taken at 97 000 magnification and defocus ‐2.0 µm.

### Cell Culture

Mouse embryonic fibroblast (MEF), NIH/3T3 (3T3), and Neuro‐2a (N2a) cells were cultured in DMEM (WELGENE) supplemented with 10% FBS (WELGENE) and 1% penicillin‐streptomycin (Thermo Fisher Scientific). Primary neurons were isolated from embryonic day 14 (E14) ICR mouse embryos and cultured in Neurobasal medium (Gibco) supplemented with FBS, B‐27 supplement (Gibco), L‐glutamine (Gibco), laminin (Sigma), and penicillin‐streptomycin. primary astrocytes were prepared from the cerebral cortices of postnatal day 0 (P0) ICR mice. Brains were sterilized with 70% ethanol and rinsed three times in PBS (Biosesang) prior to dissociation. Astrocytes were cultured in DMEM with 10% FBS and 1% penicillin‐streptomycin. For non‐contact neuron‐astrocyte co‐culture experiments, primary neurons were plated onto poly‐D‐lysine‐coated glass coverslips in 24‐well plates, while primary astrocytes were seeded into the upper chambers of Transwell inserts (SPL). This co‐culture system allowed astrocytes to sequester extracellular Aβ42 and thereby reduce its exposure to neurons, without direct cell–cell contact. Astrocytes were pre‐treated with Mt3 CRISPRa lipid nanoparticles (Mt3‐LNPs) for 48 h, after which the inserts were transferred to wells containing neurons to initiate co‐culture.

### Animal Experiments

All animal experiments were approved by the Institutional Animal Care and Use Committee at Chungbuk University and performed in accordance with institutional guidelines. ICR mice were obtained from Daehan Bio Link (Chungbuk, Korea), and 5XFAD mice were obtained from the Jackson Laboratory. Experiments were conducted on 5‐month‐old male ICR and 5XFAD mice. For stereotaxic injections, mice were anesthetized with Avertin (120 mg kg^−1^, Sigma), and 1 µL of Mt3 CRISPRa lipid nanocomplex was microinjected into the dentate gyrus of each hemisphere at the following coordinates: AP ‐2.1 mm, ML ± 1.4 mm, and DV ‐1.55 mm. After the procedure, mice were kept warm and monitored until they were fully awake. One month later, behavioral tests and biochemical analyses were conducted with mice (*n* = 5 per group). Behavioral assessments included the water maze, Y‐maze, and contextual fear conditioning tests. In the water maze test, mice were trained in a circular pool with a visible platform for 4 days (3 trials per day). On the fifth day, the invisible platform test was conducted, and the time spent in each quadrant over 1 min was recorded. The Y‐maze test assessed spontaneous alternation behavior in a three‐arm maze, where mouse movements were tracked for 10 min, and the total number of arm entries was counted. The contextual fear conditioning test was performed over two days. On the first day, mice explored the conditioning chamber for 3 min, followed by exposure to paired conditioned and unconditioned aversive stimuli (1 s, 0.7 mA). On the second day, freezing behavior was recorded for 2 min to evaluate conditioned fear responses. All behavioral tests were recorded and analyzed using Noldus Ethovision XT 13 (Noldus). Mice were randomly assigned to behavioral assessments, and all procedures were performed in a blinded manner. Following behavioral testing, mice were sacrificed for subsequent biochemical analysis. Both behavioral and histological analyses were conducted independently.

### Western Blot Analysis

For western blot, samples were lysed using RIPA buffer (Sigma) supplemented with a 1 × protease inhibitor cocktail (GenDEPOT) and 5 × loading buffer. The lysates were heated at 95 °C for 10 min and then centrifuged at 14 000 × g for 10 min to remove any debris. The resulting supernatants were subjected to SDS‐PAGE and transferred onto membranes. The membranes were incubated overnight at 4 °C with primary antibodies specific for Metallothionein 3 (Bioss, BS‐4940R), Clathrin Heavy Chain (Cell Signaling, 4796S), and β‐actin (Santa Cruz Biotechnology, sc‐47778). Subsequently, the blots were incubated with secondary antibodies for 1 h at room‐temperature. Visualization was performed using the ECL detection kit (Cytiva).

### Immunocytochemistry

Cells were first fixed in 4% paraformaldehyde (Sigma) and then rinsed in phosphate‐buffered saline (PBS). After fixation, samples were incubated in PBST (PBS containing 0.1% Tween‐20) with 1% bovine serum albumin (BSA) for 20 min to block non‐specific binding. The samples were then exposed overnight at 4 °C to primary reagents anti‐GFAP (Invitrogen, #13‐0300), Clathrin Heavy Chain (Cell Signaling, 4796S), Albumin from Bovine Serum (BSA)‐ Alexa Fluor 488 (Invitrogen, A13100) and Phalloidin‐iFluor 488 (Abcam, ab176753) to label astrocytes. After washing with 0.1% PBST, appropriate secondary antibodies were applied for 2 h at room‐temperature. Nuclear staining was performed with 4′,6‐diamidino‐2‐phenylindole (DAPI; Invitrogen). Images were captured using a Zeiss LSM 700 confocal microscope, and quantification was performed with ImageJ (NIH) software. The average number or intensity of all samples in each group was quantified, using data from at least three wells or slices. The experimenter was not blind to the treatment conditions, and no cell cultures were excluded from the analysis.

### RNA Isolation and qRT‐PCR

Total RNA was isolated using the eCube Tissue RNA Mini Kit (Philekorea) according to the manufacturer's guidelines. Briefly, 1 µg of RNA was used for reverse transcription to synthesize cDNA with AccuPower CycleScript RT PreMix (Bioneer). Quantitative real‐time polymerase chain reaction (qRT‐PCR) qRT‐PCR was performed using a QuantStudio1 Real‐Time PCR machine (Applied Biosystems) using specific primers and RealAmp 2X qPCR Master Mix, High Rox (Geneall) for amplification.

### Preparation of Aβ Peptides

Synthetic human Aβ_1_₋_42_ peptides (MedChemExpress) were first dissolved in 1,1,1,3,3,3‐hexafluoro‐2‐propanol (HFIP; Sigma) to monomerize the peptides. The solution was thoroughly vortexed and sonicated in a water bath sonicator. HFIP was then completely evaporated under a gentle stream of nitrogen gas, and the resulting peptide film was stored at –80 °C until use. For oligomer preparation, the dried peptide film was re‐dissolved in DMSO stock was diluted in ice‐cold PBS, and incubated at 4 °C for 24 h without agitation to allow oligomerization. Following the initiation of co‐culture, Aβ_1_₋_42_ oligomers were added to the neuronal compartment at a final concentration of 1 µm and incubated for 3 days.

### Aβ42 and Aβ40 Quantification

Cell and brain tissue samples were homogenized using RIPA buffer (Sigma) supplemented with a 1 × protease inhibitor cocktail (GenDEPOT) to prevent protein degradation. The concentrations of Aβ40 and Aβ42 were then determined with ELISA kits specific for Aβ (1‐40) and Aβ (1‐42) (FL) from IBL International. Finally, absorbance was measured at 450 nm using a SpectraMax i3x reader (Molecular Devices).

### Off‐Targets Analysis

Off‐target sequences were identified via Cas‐OFFinder (http://www.rgenome.net/cas‐offinder), an off‐target site prediction software. The five most probable off‐target sites, with no more than three mismatches from the intended target sequence, were confirmed using quantitative real‐time PCR.

### Thioflavin T (ThT) Staining

The ThT staining was performed on brain samples by incubating them in a 50% ethanol solution containing ThT for 10 min. Following staining, the samples were thoroughly washed with PBS to remove excess dye. To visualize cell nuclei, the samples were counterstained with DAPI before imaging. Confocal images were acquired using a Zeiss LSM 700 microscope (Carl Zeiss). Images were quantified with ImageJ (NIH) software.

### scRNA‐seq Data Acquisition

scRNA‐seq datasets previously generated and published by our group (Accession: GSE224398) were used for this study. Raw sequencing data were processed as described in the previous work.^[^ ^23^ ^]^

### Statistical Analysis

GraphPad Prism 9.3.1 software was used to perform statistical analyses. Group differences were evaluated through one‐way or two‐way ANOVA and two‐tailed unpaired *t*‐tests. Statistical significance was defined as a *p*‐value of < 0.05.

## Conflict of Interest

The authors declare no conflict of interest.

## Author Contributions

J.P. and B.K. contributed equally to this work. J.P. and B.K. performed experiments and data analysis; M.H., M.P., H.R., and H.Y. performed experiments; C.‐W.P. contributed to data interpretation; S.‐B.Y. performed experiments, analyzed data, interpreted results, and wrote the manuscript; H.P. conceived the study, interpreted data, and wrote the manuscript.

## Supporting information



Supporting Information

## Data Availability

The data that support the findings of this study are available from the corresponding author upon reasonable request.
