# Supplementary material for: CRISPRa Lipid Nanocomplex‐Mediated Mt3 Targeting Enhances Astrocytic Endocytosis of Amyloid‐β in an Alzheimer's Disease Mouse Model
Source: Adv Sci (Weinh). 2025 Dec 12;13(4):e03725. doi: 10.1002/advs.202503725 (PMC12822419; doi:10.1002/advs.202503725)
Supplement: Supplementary file 1 — Supporting Information [file ADVS-13-e03725-s001.docx]

Supporting Information

**CRISPRa Lipid Nanocomplex-Mediated Mt3 Targeting Enhances Astrocytic Endocytosis of Amyloid-β** **in an Alzheimer’s Disease Mouse Model**

*Junhang Park^1,5^, Boyoung Kim^1,5^, Minki Ha^2^, Moonsu Park^1^, Hongji Ryu^1^, Hyerin Yu^1^, Sungsoo Park^4^,* *Yoon-Seok Roh*^3^*, Key-Hwan Lim*^3^*, Jin Tae Hong*^3^*, Sang-Bae Han*^3^*, Chun-Woong Park*^3^**, Seok-Beom Yong^2^*, Hanseul Park^1^**

^1^ Laboratory of Molecular Genetics, College of Pharmacy, Chungbuk National University, Cheongju, 28160, Republic of Korea

^2^ Center for Gene & Cell Therapy, Korea Research Institute of Bioscience and Biotechnology (KRIBB), Chungcheongbuk-do, 28116 Republic of Korea

^3^ College of Pharmacy, Chungbuk National University, Osongsaengmyeong 1-ro, Osong-eup, Heungdeok-gu, Cheongju, 28160, Republic of Korea.

^4^ College of Veterinary Medicine, Jeonbuk National University, Iksan 54596, Republic of Korea

^5^ These authors contributed equally

*Co-Correspondence:

Hanseul Park, Ph.D.

Laboratory of Molecular Genetics

College of Pharmacy

Chungbuk National University, Cheongju, Republic of Korea

Email: hanpark@chungbuk.ac.kr

Seok-Beom Yong, Ph.D.

Korea Research Institute of Bioscience and Biotechnology (KRIBB)

Email: sbyong@kribb.re.kr

Chun-Woong Park, Ph.D.

College of Pharmacy, Chungbuk National University, Cheongju, Republic of Korea

Email: cwpark@chungbuk.ac.kr

**
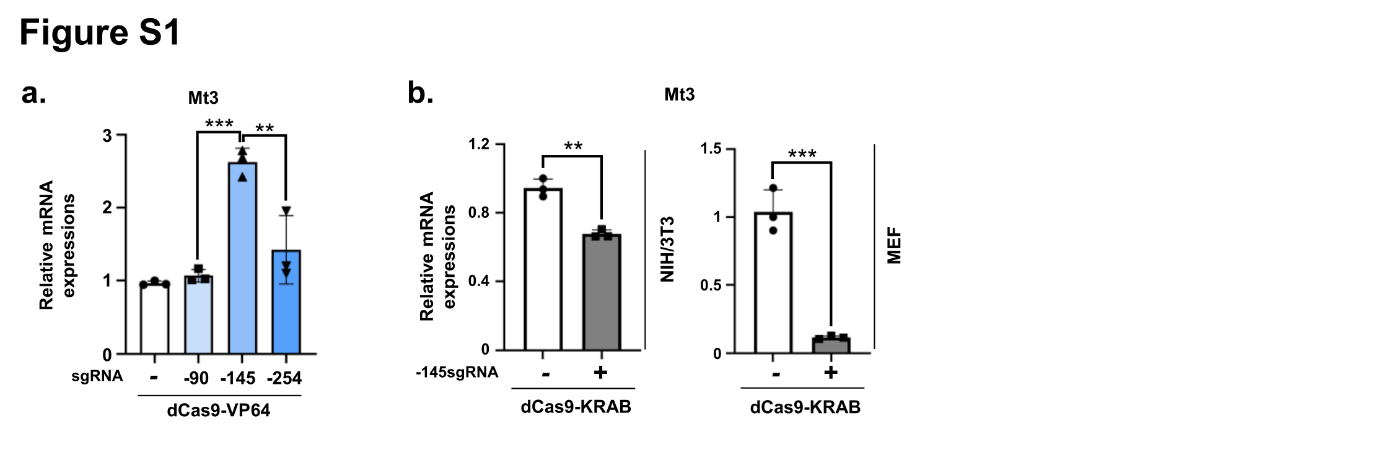
**

**Figure S1. CRISPRa and CRISPRi modulation of Mt3 expression *in vitro*.** (a) Quantitative real-time PCR analysis of Mt3 mRNA expression in mouse primary astrocytes treated with sgRNAs targeting −90, −145, and −254 bp upstream of the Mt3 gene start codon. Data are expressed as mean  ±  SEM (n  =  3). ***p*  <  0.01, ****p*  <  0.001, one-way ANOVA with Tukey’s multiple comparisons test. (b) Quantitative real-time PCR analysis of Mt3 mRNA expression in NIH/3T3 cells and MEF cells treated with −145 bp sgRNA and dCas9-KRAB. Data are expressed as mean  ±  SEM (n  =  3). ***p*  <  0.01, ****p*  <  0.001, two-sided Student’s t-test.


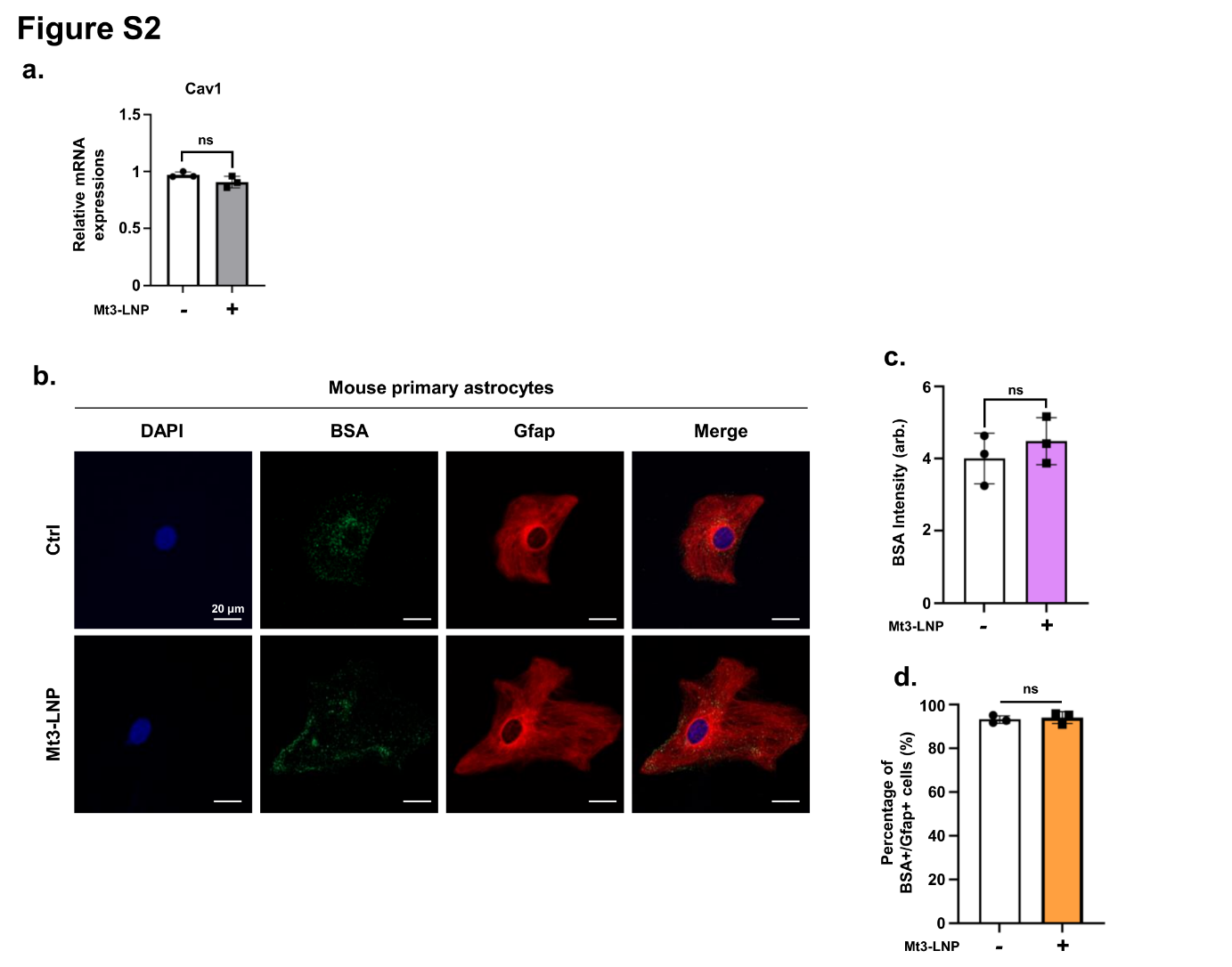

**Figure S2. Mt3 activation enhances clathrin-mediated endocytosis in astrocytes.** (a) Quantitative real-time PCR analysis of Cav1 gene expression in mouse primary astrocytes treated with Mt3 CRISPRa lipid nanocomplexes. Data are expressed as mean  ±  SEM (n  =  3). **p*  <  0.05, two-sided Student’s t-test. (b) Immunostaining of mouse primary astrocytes with BSA (green), Gfap (red), and DAPI (blue) following a 2 day treatment with Mt3 CRISPRa lipid nanocomplexes, 30 min exposure to Alexa Fluor488-BSA. (c) Quantification of BSA intensity from Figure S2b. Data are expressed as mean  ±  SEM (n  =  3 biological replicates, with > 50 Gfap-positive cells analyzed per group). **p*  <  0.05, two-sided Student’s t-test. (d) Percentage of BSA/Gfap double-positive cells from Supplementary Figure 2b. Data are expressed as mean  ±  SEM (n  =  3 biological replicates, with > 50 Gfap-positive cells per group). **p*  <  0.05, two-sided Student’s t-test. The images in (b) are representative of three or more independent experiments.


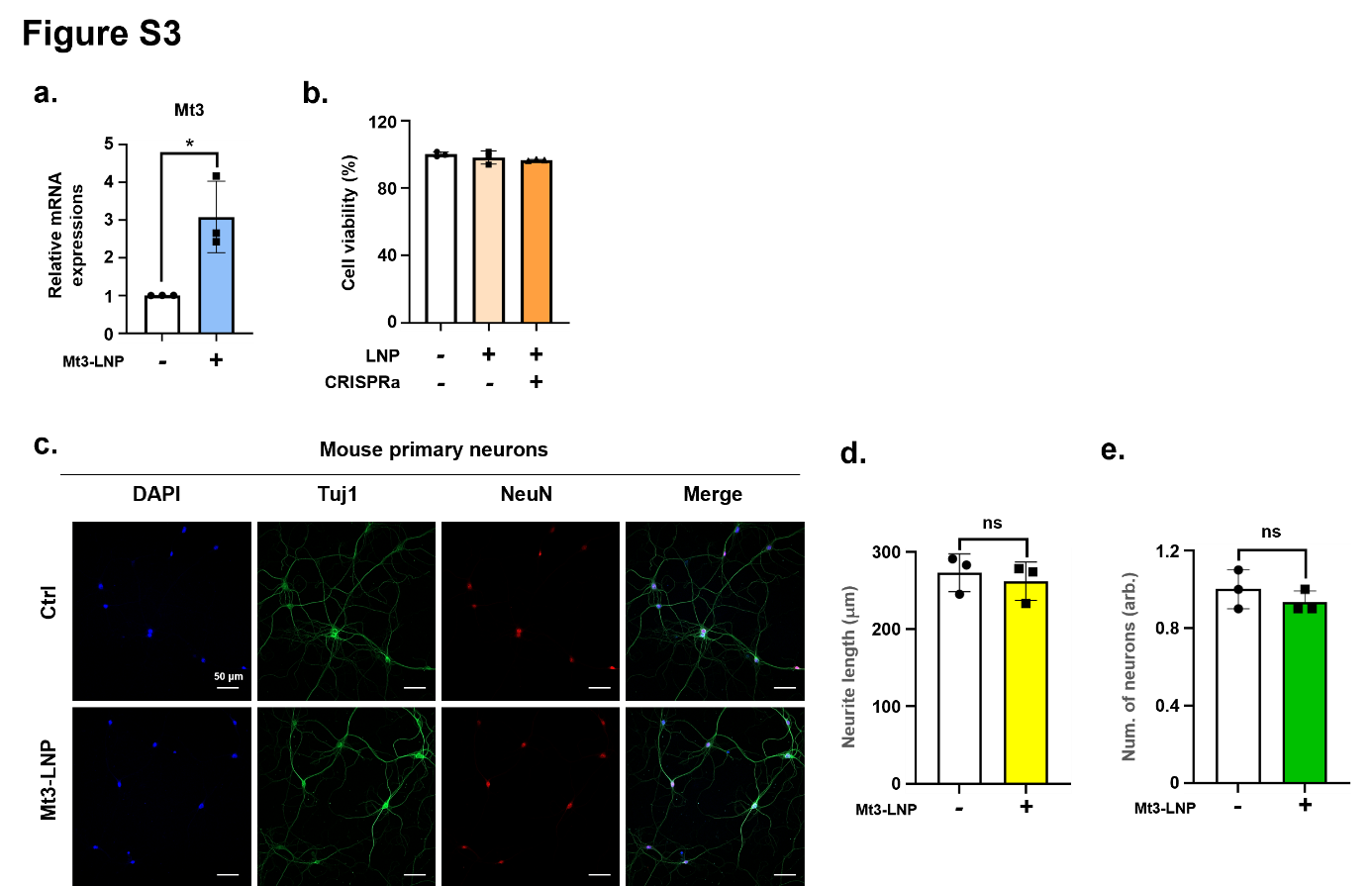


**Figure S3. Neuronal safety evaluation of Mt3 CRISPRa lipid nanocomplexes.** (a) Quantitative real-time PCR analysis of Mt3 gene expression in mouse primary neurons treated with Mt3 CRISPRa lipid nanocomplexes. Data are expressed as mean  ±  SEM (n  =  3). *p  <  0.05, two-sided Student’s t-test. (b) MTT assay of mouse primary neurons viability after treatment with Empty-LNP or Mt3 CRISPRa lipid nanocomplexes at 2 µg for 48 h. Data are expressed as mean  ±  SEM (n  =  3). **p*  <  0.05, two-way ANOVA with Tukey’s multiple comparisons test. (c) Immunostaining for Tuj1 (green), NeuN (red), and DAPI (blue) in mouse primary neurons. (d) Quantification of Tuj1-positive cells from Figure S3c. Data are expressed as mean  ±  SEM (n  =  3). **p*  <  0.05, two-sided Student’s t-test. (e) Quantification of neurite length in primary neurons. Data are expressed as mean  ±  SEM (n  =  3 biological replicates, with > 100 Tuj1/Neun double-positive cells per group). **p*  <  0.05, two-sided Student’s t-test. The images in (c) are representative of three or more independent experiments.


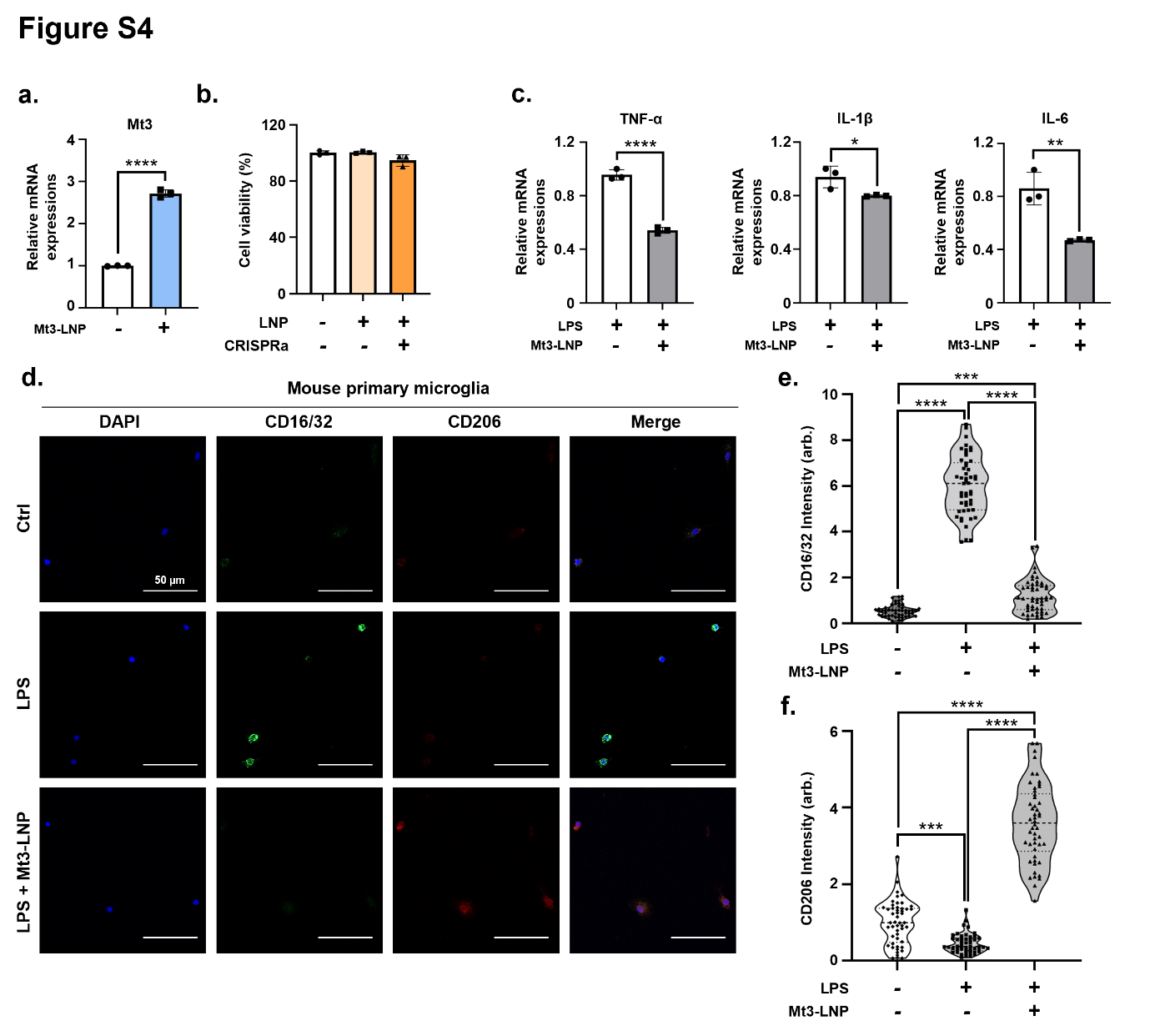

**Figure S4. Mt3 activation promotes anti-inflammatory polarization in microglia.** (a) Quantitative real-time PCR analysis of Mt3 gene expression in mouse primary microglia treated with Mt3 CRISPRa lipid nanocomplexes. Data are expressed as mean  ±  SEM (n  =  3). *****p*  <  0.0001, two-sided Student’s t-test. (b) MTT assay of mouse primary microglia viability after treatment with Empty-LNP or Mt3 CRISPRa lipid nanocomplexes at 2 µg for 48 h. Data are expressed as mean  ±  SEM (n  =  3). **p*  <  0.05, two-way ANOVA with Tukey’s multiple comparisons test. (c) Quantitative real-time PCR analysis of TNF-α, IL-1β, and IL-6 gene expression in mouse primary microglia treated with Mt3 CRISPRa lipid nanocomplexes, following a 2 day treatment with Mt3 CRISPRa lipid nanocomplexes and a subsequent 1 day exposure to LPS. Data are expressed as mean  ±  SEM (n  =  3). **p*  <  0.05, ***p*  <  0.01, *****p*  <  0.0001, two-sided Student’s t-test. (d) Immunostaining of mouse primary microglia with CD16/32 (green), CD206 (red), and DAPI (blue) following a 2 day treatment with Mt3 CRISPRa lipid nanocomplexes, 1 day exposure to LPS. (e) Quantification of CD16/32 intensity from Supplementary Figure 4d. Data are expressed as mean  ±  SEM (n  =  50). ****p*  <  0.001, *****p*  <  0.0001, two-way ANOVA with Tukey’s multiple comparisons test. (f) Quantification of CD206 intensity from Supplementary Figure 4d. Data are expressed as mean  ±  SEM (n  =  50). ***p  <  0.001, ****p  <  0.0001, two-way ANOVA with Tukey’s multiple comparisons test.


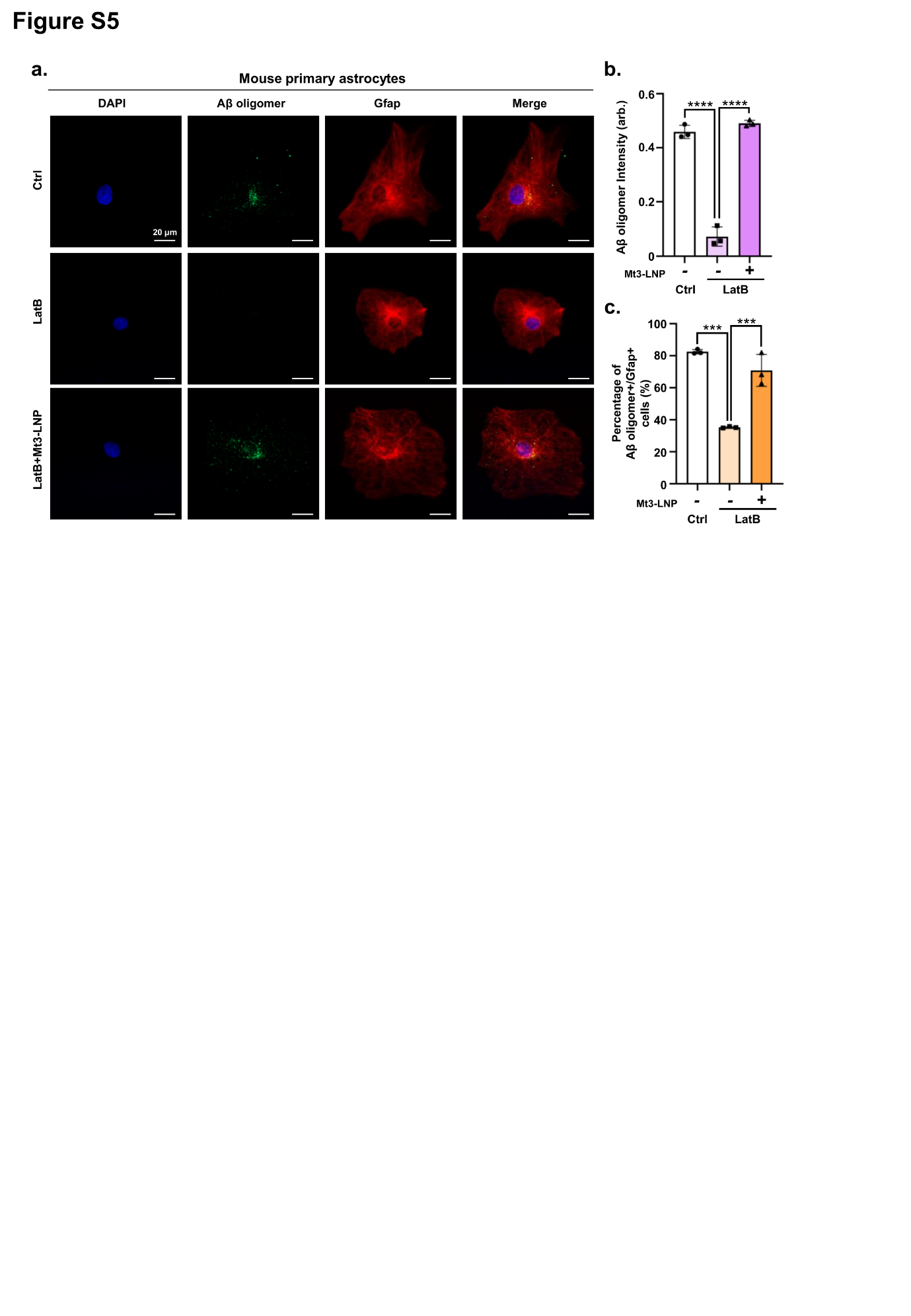

**Figure S5. Mt3 activation facilitates Aβ oligomer uptake in astrocytes.** (a) Immunostaining of mouse primary astrocytes with Aβ oligomer (green), Gfap (red), and DAPI (blue) following a 2 day treatment with Mt3 CRISPRa lipid nanocomplexes, 1 h LatB treatment, and 15 min exposure to Aβ oligomer. (b) Quantification of Aβ oligomer intensity from Supplementary Figure 5a. Data are expressed as mean  ±  SEM (n  =  3 biological replicates, with > 50 Gfap-positive cells analyzed per group). *****p*  <  0.0001, two-way ANOVA with Tukey’s multiple comparisons test. (c) Percentage of Aβ oligomer/Gfap double-positive cells from Supplementary Figure 5a. Data are expressed as mean  ±  SEM (n  =  3 biological replicates, with > 50 Gfap-positive cells per group). ****p*  <  0.001, two-way ANOVA Tukey’s multiple comparisons test. Over 100 astrocytes were analyzed. The images in (a) are representative of three or more independent experiments.


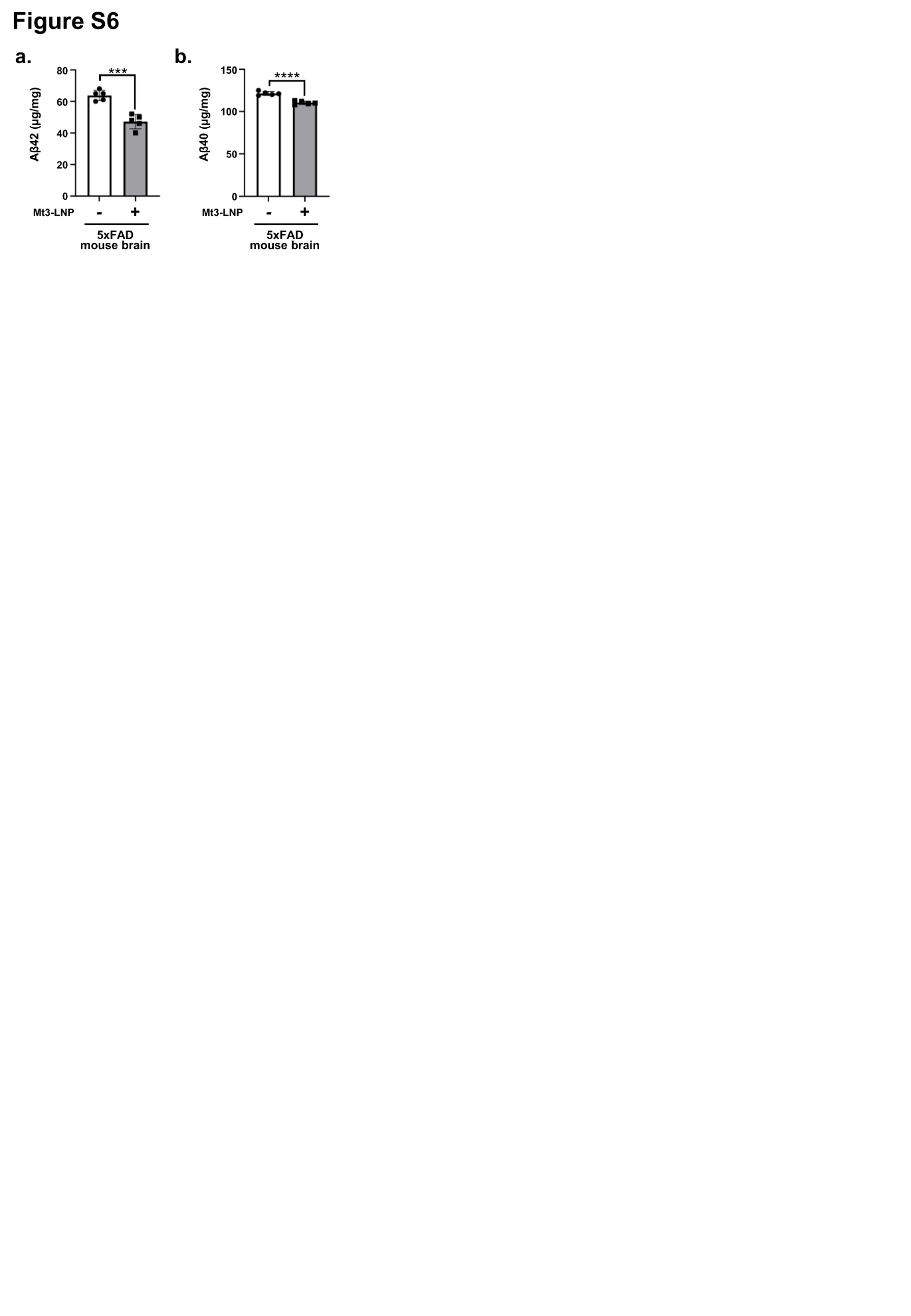


**Figure S6. Reduction of Aβ levels following Mt3 CRISRPa treatment.** (a) ELISA analysis of Aβ42 levels in the brains of 5xFAD mice injected with Mt3 CRISPRa lipid nanocomplex. Data are presented as mean ± SEM (n = 5). ***p < 0.001, two-sided Student’s t-test. (b) ELISA analysis of Aβ40 levels in the brains of 5xFAD mice injected with Mt3 CRISPRa lipid nanocomplex. Data are expressed as mean ± SEM (n = 5). ****p < 0.0001, two-sided Student’s t-test.


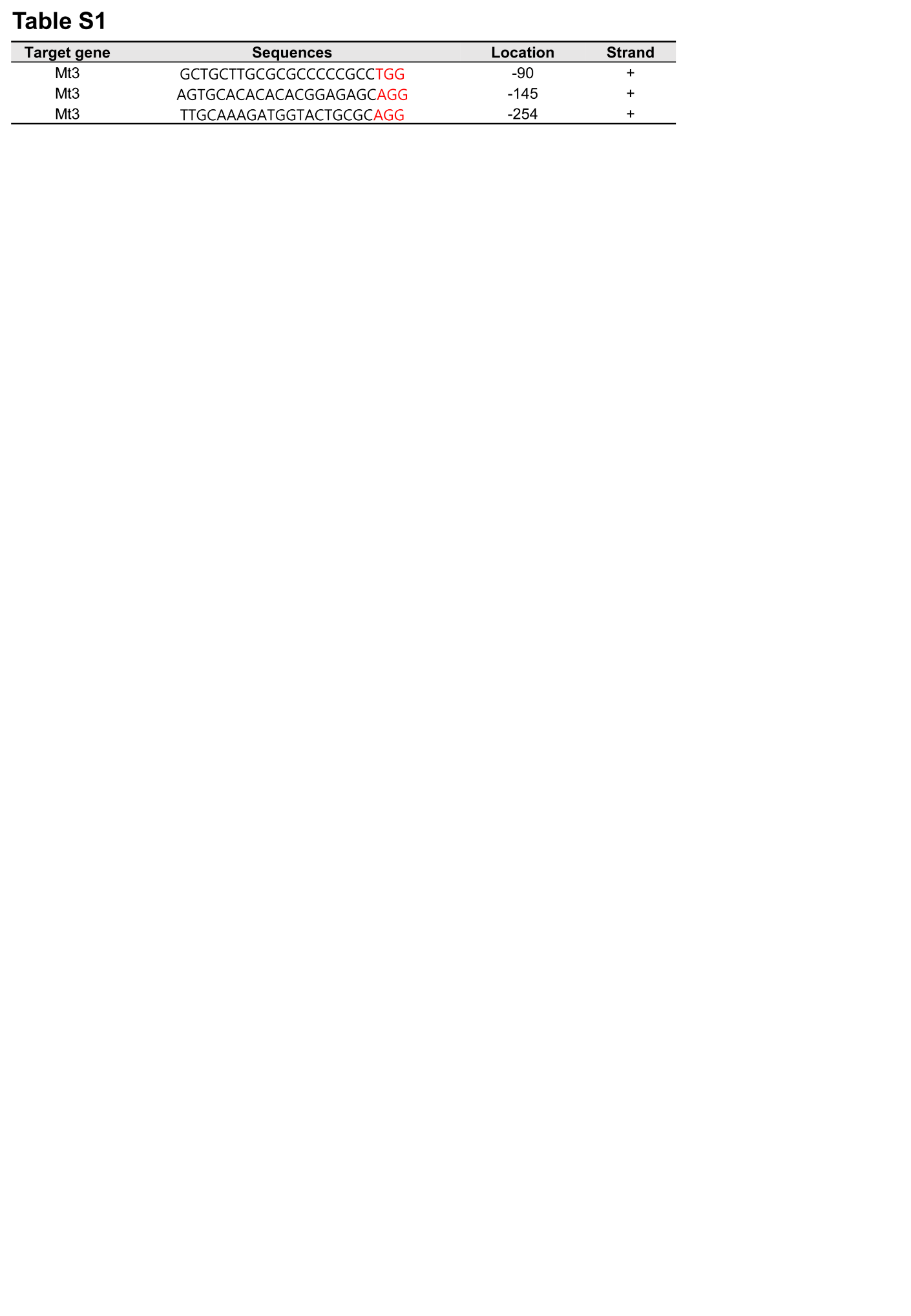


**Table S1. sgRNA sequences targeting Mt3. Protospacer adjacent motif sequences are underlined in red.**

**
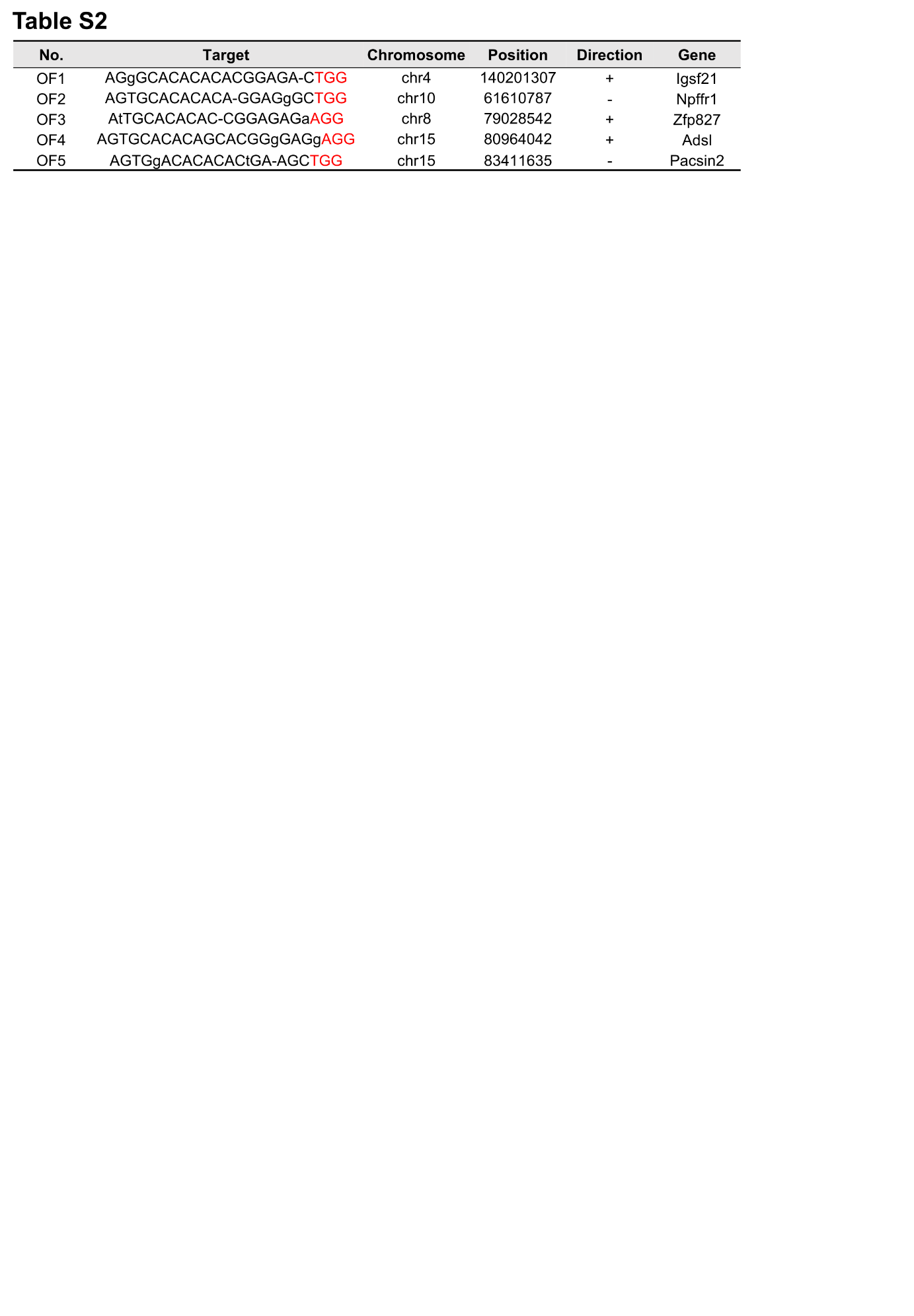

Table S2. Predicted Mt3 off-target sites for −145 sgRNA sequence.** Off-target sites were predicted based on sequences with no more than three mismatches compared to the on-target sequence. Abbreviation: OF, off-target.
